# Associations of hyperthyroidism with epilepsy: a Mendelian randomization study

**DOI:** 10.1038/s41598-024-54933-w

**Published:** 2024-02-27

**Authors:** Jinwen Liu, Han Yu, Qin Wang, Jie Zhong, Chunyuan Yao, Jiangwei Chen, Limei Diao

**Affiliations:** 1 Guangxi University of Chinese Medicine, Nanning, 530200 China; 2 Harbin Medical University, Harbin, China; 3 The First Affiliated Hospital of Guangxi University of Chinese Medicine, Guangxi University of Chinese Medicine, Nanning, 530023 China

**Keywords:** Hyperthyroidism, Epilepsy, Mendelian randomization, Geno, Computational biology and bioinformatics, Genetics, Neuroscience, Endocrinology, Medical research, Neurology

## Abstract

Prior studies have revealed an increased susceptibility to epilepsy in hyperthyroid individuals, but the genetic basis of the hyperthyroidism–epilepsy relationship is not fully comprehended, prompting this study to explore this potential association. We conducted a two-sample Mendelian randomization (TSMR) study to explore the relationship between hyperthyroidism and epilepsy by utilizing aggregated statistics from Genome-Wide Association Studies (GWAS). Data for hyperthyroidism were derived from a GWAS encompassing 462,933 participants, while epilepsy data were sourced from the International League Against Epilepsy (ILAE) consortium. Five distinct methods were employed for TSMR analysis, which included the inverse variance weighting method, MR Egger method, weighted median method, simple model, and weighted model. In our sensitivity analysis, we employed the MR Egger and MR PRESSO methods to assess pleiotropy, and inverse variance weighting and MR Egger in Cochran’s Q statistics to assess heterogeneity. In the IEU database, utilizing the MR-Egger method, we obtained an odds ratio (OR) of 2.631 (95% CI 0.608, 9.796) with a p-value of 0.122. Meanwhile, employing the Weighted Median method yielded an OR of 1.813 (95% CI 0.786, 4.181) with a p-value of 0.163. The IVW method exhibited an OR of 1.986 (95% CI 1.127, 3.502) with a p-value of 0.018. In the assessment of heterogeneity, the MR-Egger method produced a Q statistic of 65.205, accompanied by a p-value of 0.087, while the IVW method recorded a Q statistic of 66.668 with a p-value of 0.083. The multifactorial analysis results showed an intercept term with a standard error (SE) value of 0.009 and a p-value of 0.291. In the FinnGen database, employing the MR-Egger method for all epilepsy data, we observed an OR of 0.952 (95% CI 0.831, 1.093) with a p-value of 0.539. Simultaneously, the Weighted Median method produced an OR of 0.986 (95% CI 0.953, 1.021) with a p-value of 0.423. The IVW method indicated an OR of 0.992 (95% CI 0.965, 1.019) with a p-value of 0.541. The MR-Egger method’s assessment of heterogeneity resulted in a Q statistic of 2.671, associated with a p-value of 0.445, while the IVW method generated a Q statistic of 3.011 with a p-value of 0.556. The multifactorial analysis results displayed an intercept term with a SE-value of 0.019 and a p-value of 0.601. Sensitivity analysis found no evidence of horizontal pleiotropy or heterogeneity. Hyperthyroidism was found to be causally related to all epilepsy but had no effect on other types of epilepsy.

## Introduction

Epilepsy is a prevalent neurological disorder characterized by sudden abnormal brain discharges^1^. It impacts over 70 million people worldwide^2^. This condition leads to substantial economic challenges and a compromised quality of life^3^.

Hyperthyroidism, or thyrotoxicosis, is a common endocrine disorder with a rising incidence and a tendency for an earlier onset^4,5^. Thyroid hormones profoundly influence the development and function of the neuromuscular system^6^. Neurological complications are common in individuals with hyperthyroidism, and some present with symptoms like tremors when seeking medical attention. Neurological complications in hyperthyroidism encompass central nervous system (CNS) issues like movement disorders, corticospinal tract damage, epilepsy, emotional and cognitive impairments, cerebrovascular diseases, migraines, and sleep disorders, along with peripheral nervous system problems like tremors, myopathy, and peripheral nerve damage. These complications mainly stem from direct stimulatory effects of thyroid hormones, a hypermetabolic state, autoimmune factors, thyroid tissue enlargement, and compression of surrounding nerves by the extraocular muscles^7^. The incidence and severity of these neuromuscular complications may vary, often correlating with hyperthyroidism severity, and typically show marked improvement with antithyroid treatment^8^.

Thyroid disorders often present with reversible neurological manifestations affecting both the central and peripheral nervous systems, including seizures, as evidenced by idiopathic seizure reports in thyrotoxicosis patients^9^. Furthermore, experimental studies involving thyroxine administration in animal models of epilepsy suggest a reduction in seizure threshold, while thyroidectomy appears to confer protection against pharmacologically induced seizures^10–12^. Presently, meta-analyses provide insights into the potential effectiveness of thyroidectomy in reducing seizure recurrence^13^. Nevertheless, the precise relationship between thyroid hyperactivity and seizure risk remains enigmatic. The disparity in research findings can be attributed to the inherent complexity of observational studies, characterized by a multitude of confounding factors and potential reverse causality. The limitations of traditional statistical methods exacerbate these intricacies, posing challenges in precisely disentangling latent confounding factors or reverse causal links, thus impeding the establishment of a robust causal framework for the observed associations^14^.

Mendelian randomization (MR) appears to be a potential route in the effort to investigate the causal association between hyperthyroidism and epilepsy while overcoming confounding factors^15–17^. MR mimics the design of randomized controlled trials (RCTs) by utilizing instrumental variables (IVs) obtained from genetic polymorphisms, allowing for a more thorough evaluation of causation^16^. The primary aim of this research endeavor is to explore the causal nexus between hyperthyroidism and epilepsy. To undertake this inquiry, we employ two-sample Mendelian randomization (TSMR) analysis, leveraging summary-level data derived from Genome-Wide Association Studies (GWAS) to expound upon this intricate interconnection.

## Materials and methods

### Study design and data sources

This study employs the TSMR approach to investigate the impact of thyroid hyperactivity on epilepsy. Data on genetic variants associated with thyroid hyperactivity as an exposure variable were sourced from the MRC-IEU, encompassing 462,933 individuals of European lineage.

**Figure 1:**
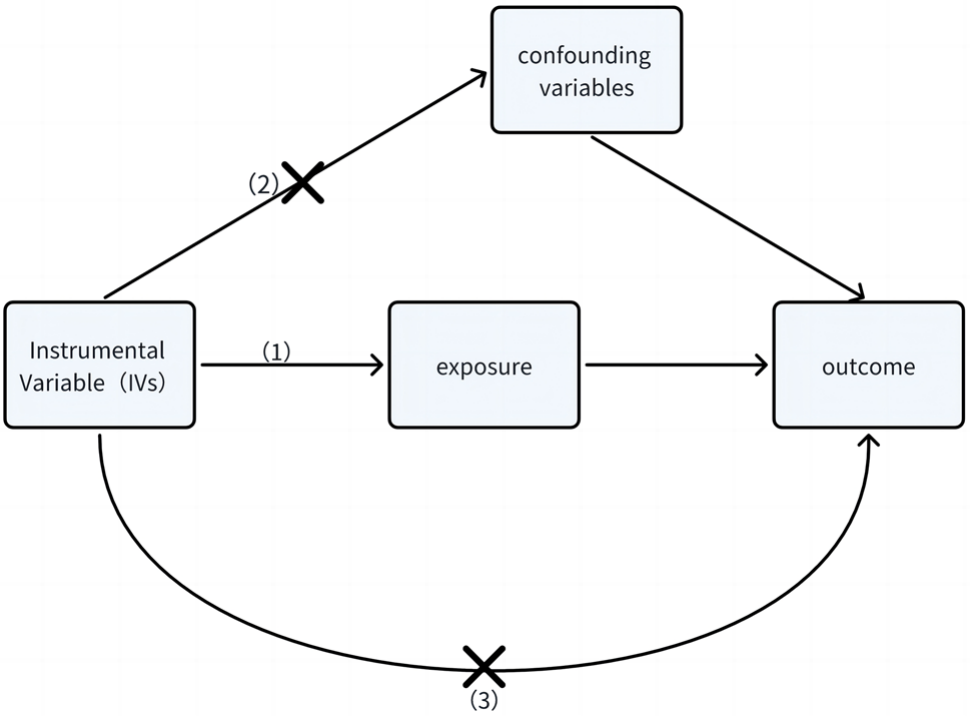
Two-sample Mendelian randomization study: (1) Strong correlation between instrumental variables and the exposure factor; (2) Independence of instrumental variables from any potential confounding factors; (3) Lack of direct association between instrumental variables and the outcome.

The dataset used for our study on genetic variations linked to epilepsy originates from the IEU OpenGWAS project and draws data from the International League Against Epilepsy Consortium on Complex Epilepsies (ILAE). This comprehensive dataset encompasses a range of epilepsy subtypes, offering valuable insights. It includes the following subtypes: (1) All epilepsy, with a substantial dataset comprising 15,212 cases and 29,677 controls. (2) Hereditary generalized epilepsy, with 3769 cases. (3) Focal epilepsy, a significant subset with 9671 cases. (4) Focal epilepsy with documented lesion absence, consisting of 2716 cases. (5) Juvenile absence epilepsy, represented by 415 cases. (6) Childhood absence epilepsy, providing insights from 793 cases. (7) Focal epilepsy with documented hippocampal sclerosis, a subset with 803 cases. (8) Focal epilepsy with documented lesions other than hippocampal sclerosis, offering data from 3070 cases. (9) Generalized epilepsy with tonic–clonic seizures, a smaller subset comprising 228 cases. (10) Juvenile myoclonic epilepsy, with data available from 1181 cases^18^. This rich and diverse dataset allows for a comprehensive exploration of genetic factors associated with epilepsy across various subtypes, providing a robust foundation for our research (Fig. 1, Supplementary Table S1).

To assess the robustness of our MR estimations, we further employed an additional GWAS dataset, wherein the source of exposure data emanated from participants in the FinnGen (n = 473,681, European ancestry) (Supplementary Table S13).

### Instrumental variable (IV) selection

By carefully selecting the genetic variations we’ve extracted as our instrumental variables, we embark on an endeavor to gauge the causal link between hyperthyroidism and the susceptibility to epilepsy. Our methodology hinges on adhering to three fundamental Mendelian randomization principles: (1) *Predictive power* We ensure that these genetic variations possess the predictive prowess to foretell the presence of hyperthyroidism, (2) *Freedom from confounding* We rigorously confirm that these genetic factors remain independent of any potential confounders that could cloud our causal assessment, (3) *Pathway integrity* We take extra precautions to ascertain that our results remain unaltered by any extraneous pathways unrelated to hyperthyroidism, thereby preserving the integrity of our causal inference framework^19^. In the initial phase of our study, we conducted a comprehensive assessment of single nucleotide polymorphisms (SNPs) associated with hyperthyroidism, subjecting them to rigorous scrutiny against a stringent genome-wide significance threshold (*P* < 5 × 10^−8^). Subsequently, we exercised prudence by excluding SNPs that demonstrated pronounced linkage disequilibrium (LD) (r^2^ < 0.001, distance < 10,000 kb) to uphold the analytical independence^20^. Furthermore, to fortify our defenses against potential pleiotropic effects, we diligently explored secondary phenotypic associations for each SNP using PhenoScanner V2^21^. The application of the F-statistic served as a stringent criterion for the exclusion of SNPs exhibiting weak instrumental properties that might compromise the validity of Mendelian Randomization’s first assumption; accordingly, only SNPs with instrument strengths (F) exceeding a threshold of 10 were retained for further analysis. Subsequently, the SNPs that remained after the initial screening were amalgamated into the outcomes Genome-Wide Association Study (GWAS) database. Palindromic SNPs characterized by intermediate allele frequencies were systematically excluded, and outlier SNPs were identified and removed through the application of the Mendelian randomization pleiotropy residual and outlier (MR PRESSO) test^20^. Notably, proxy SNPs were not utilized in this process. Adhering to the aforementioned principles, a final set of multiple independent SNPs, each strongly associated with the respective exposure trait, were meticulously chosen to serve as instrumental variables (IVs).

### Statistical analysis

Within this TSMR investigation, we employed a comprehensive suite of methodological approaches, encompassing Inverse Variance Weighting (IVW), Weighted Median (WM), MR Egger regression, Simple Mode, and Weighted Mode techniques, to conduct a rigorous assessment of the potential causal link between hyperthyroidism and epilepsy^18,20,22^. Our primary analytical framework, predicated upon the Inverse Variance Weighting (IVW) method, amalgamates Wald estimates for individual single nucleotide polymorphisms (SNPs) through a meta-analysis framework, thereby deriving a comprehensive estimation. Subsequently, it facilitates a weighted linear regression, enforcing an intercept set at zero. When executed under the fulfillment of the three fundamental instrumental variable assumptions, this method enhances precision and statistical power, culminating in an overarching estimate of the causal effect^23^. To enhance the resilience of our findings and mitigate potential sources of unmeasured confounding and unaccounted interference, we conducted supplementary analyses, employing the MR Egger regression, WM, Simple Mode, and Weighted Mode methodologies. Specifically, the MR Egger regression was utilized to scrutinize the presence of pleiotropy, incorporating an intercept term under the assumption that the strength of instrumental variables bore no correlation with the direct effect, thereby rectifying potential biases stemming from this phenomenon^22^. Should the intercept term equate to zero, this observation signifies the absence of horizontal pleiotropy. Furthermore, it is noteworthy that the weighted median (WM) method affords consistent estimations of causality, even in scenarios where up to 50% of genetic variants within the gene under consideration exhibit null effects^24^.

In the evaluation of pleiotropy among instrumental variables (IVs) within the Genome-Wide Association Study (GWAS) dataset pertaining to outcomes, we employed the MR PRESSO approach and calculated the MR Egger intercept^20^. Within the framework of these GWAS data, pleiotropy was deemed insubstantial when the p-values derived from the pleiotropy test surpassed the threshold of 0.05. To evaluate the heterogeneity of instrumental variables within the outcomes GWAS dataset, we employed the MR Egger method in conjunction with the IVW approach, incorporating Cochran’s Q statistics^25^. Heterogeneity was deemed absent if the P-value from the heterogeneity test exceeded 0.05. Furthermore, we employed scatter plots to examine the individual hypothesized causal effects.

The F-statistic was computed through the following mathematical expression: F = [R^2^(N − 2)]/[1 − R^2^], wherein R^2^ represents the proportion of variance explicated by each instrumental variable, and N signifies the sample size of the Genome-Wide Association Study (GWAS) regarding the linkage between the single nucleotide polymorphism (SNP) and the variable of interest. The determination of R^2^ was achieved through the subsequent mathematical formula: R^2^ = 2 × EAF × (1 − EAF) × β^2^, where EAF indicates the frequency of the effect allele, and β denotes the estimated effect magnitude on the variable. When the associated F-statistic exceeded P > 10, it indicated the absence of significant weak instrument bias.

Statistical analyses were conducted utilizing R version 4.2.1, as provided by the R Foundation for Statistical Computing. Mendelian Randomization (MR) analyses were executed employing both the two-sample MR (version 0.5.6) and MRPRESSO (version 1.0) methodologies^18,20,26^.

## Results

### Results of the analysis of all epilepsy

**Figure 2:**
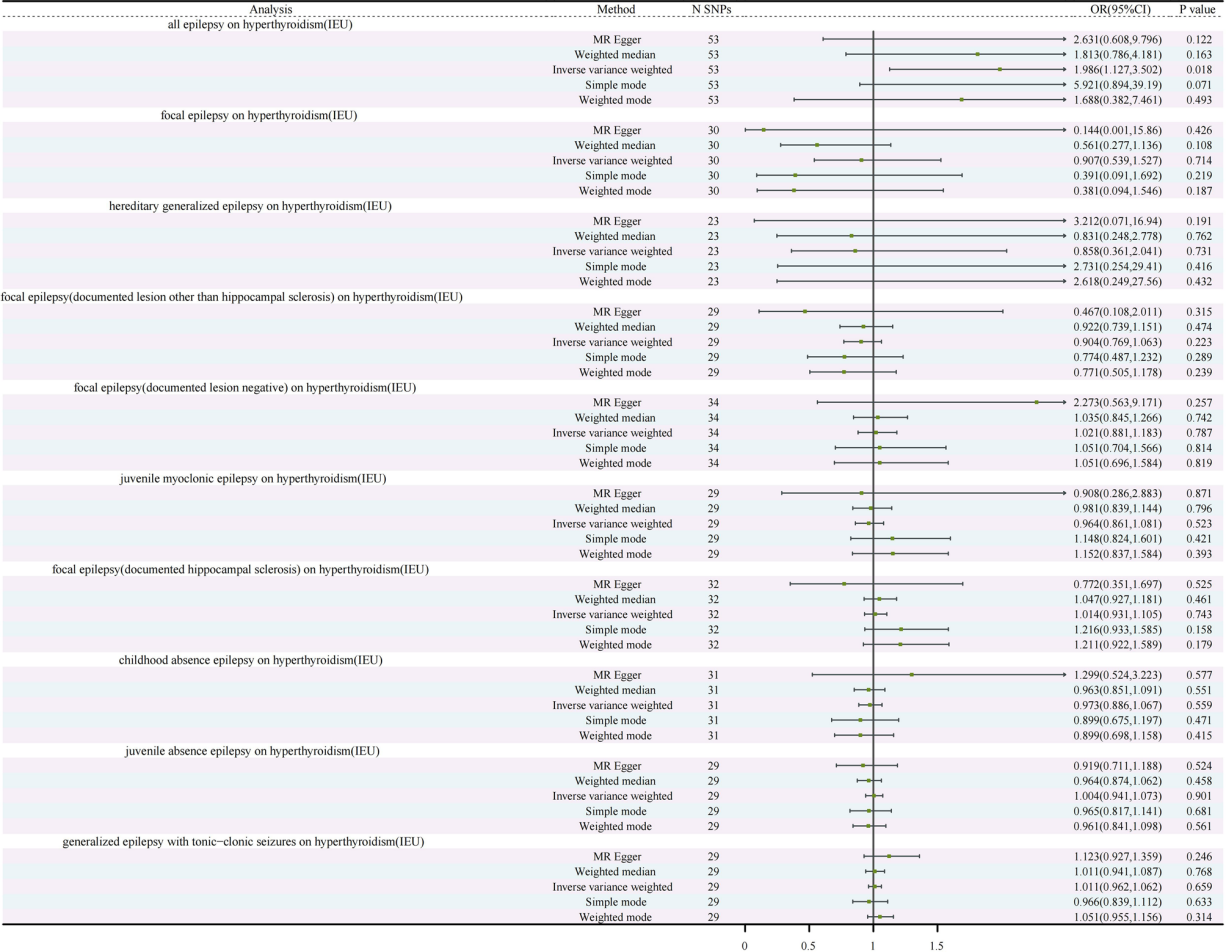
Forest plot of the genetic causal relationship between hyperthyroidism (IEU) and epilepsy. *CI* confidence interval, *N* number, *OR* odds ratios, *SNP* single-nucleotide polymorphism.

Following a meticulous and discerning selection process, we judiciously chose a total of 53 SNPs as instrumental variables for inclusion in the study of hyperthyroidism. The results of Mendelian randomization consistently exhibit coherence across a range of analytical methodologies, including the Inverse Variance Weighted (IVW) method, MR-Egger method, Weighted Median (WM) method, Simple Mode method, and Weighted Mode method. Specifically, IVW results reveal an odds ratio (OR) of 1.986 (95% CI 1.127, 3.502) with a p-value of 0.018, signifying a robust correlation between all epilepsy and hyperthyroidism. It’s noteworthy that the Q-value for the IVW heterogeneity test is 66.668 with a p-value of 0.083, while the Q-value for the MR-Egger heterogeneity test is 65.205 with a p-value of 0.087, thus firmly establishing the absence of heterogeneity. Sensitivity analysis demonstrates that, in the context of the one-by-one exclusion approach, no SNPs exert a significant influence on the estimates of causal association. Furthermore, the results of gene pleiotropy analysis indicate an intercept term with a standard error(SE) value of 0.009 and a p-value of 0.291, once again confirming the absence of horizontal pleiotropy (Fig. 2, Supplementary Table S3, Supplementary Fig. 1).

**Figure 3:**
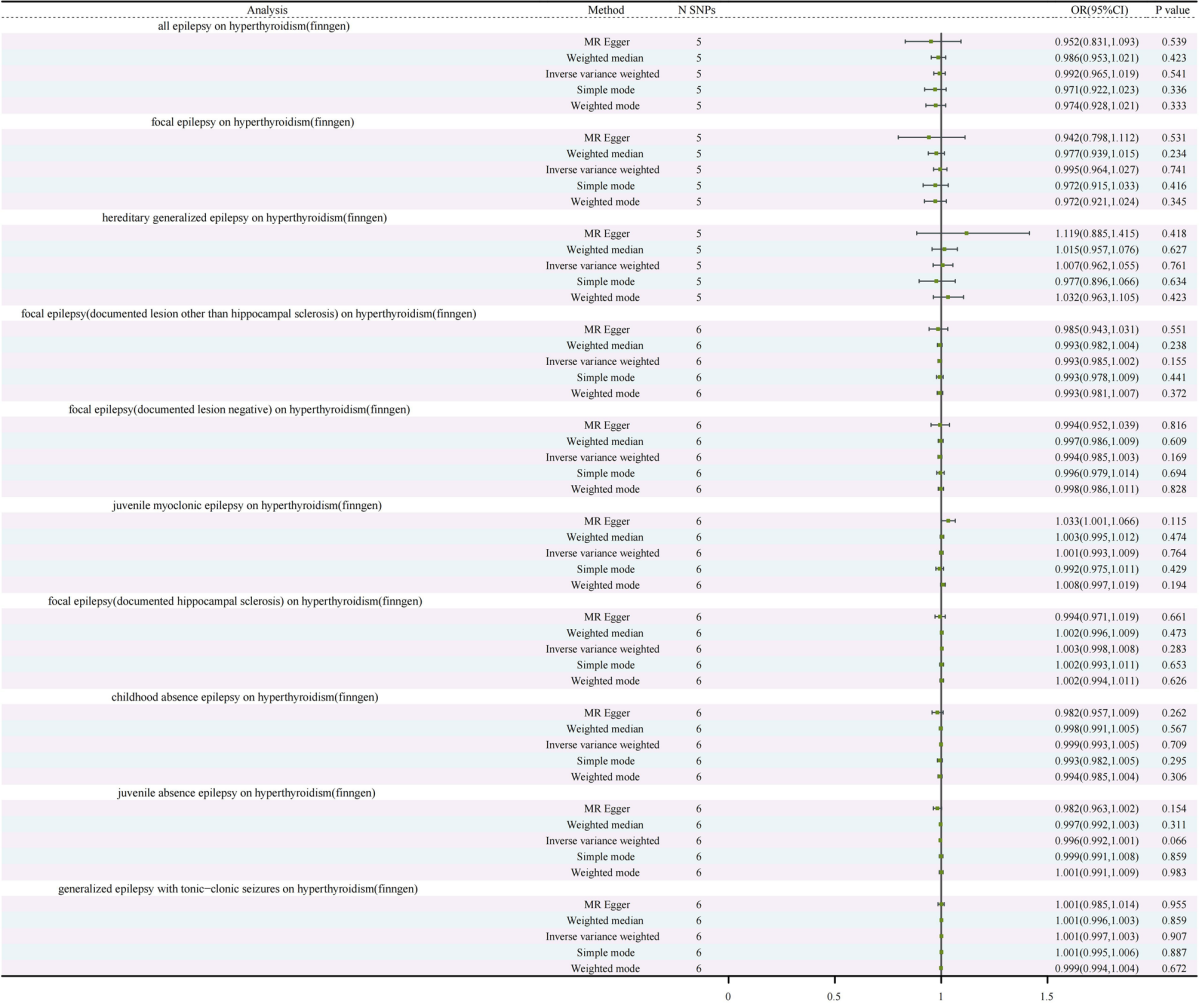
Forest plot of the genetic causal relationship between hyperthyroidism (FinnGen) and epilepsy. *CI* confidence interval, *N* number, *OR* odds ratios, *SNP* single-nucleotide polymorphism.

To assess the elasticity of our MR estimates, we followed a rigorous selection process, incorporating five SNPs from the FinnGen database as instrumental variables for hyperthyroidism. Mendelian randomization was conducted on the dataset, once again utilizing the IVW method, MR-Egger method, WM method, Simple Mode method, and Weighted Mode method for analysis. Specifically, IVW results reveal an odds ratio of 0.992 (95% CI 0.965, 1.019) with a p-value of 0.541, indicating the absence of a substantial correlation between all epilepsy and hyperthyroidism. Furthermore, the Q-value for the IVW heterogeneity test is 3.011 with a p-value of 0.556, while the Q-value for the MR-Egger heterogeneity test is 2.671 with a p-value of 0.445, confirming the absence of heterogeneity. Sensitivity analysis demonstrates that, within the context of the one-by-one exclusion method, no SNPs significantly impact the estimates of causal association. Additionally, the results of gene pleiotropy analysis indicate an intercept term with a SE-value of 0.019 and a p-value of 0.601, consistently affirming the absence of horizontal pleiotropy (Fig. 3, Supplementary Table S15, Supplementary Fig. 11).

### Lack of correlations between hyperthyroidism and various epilepsy

In the comprehensive Mendelian randomization analysis exploring potential associations between hyperthyroidism and diverse epilepsy subtypes such as focal epilepsy, hereditary generalized epilepsy, focal epilepsy with lesions other than hippocampal sclerosis, focal epilepsy with negative lesions, juvenile myoclonic epilepsy, focal epilepsy with hippocampal sclerosis, childhood absence epilepsy, juvenile absence epilepsy, and generalized epilepsy with tonic–clonic seizures, a prevalent finding emerged: the absence of significant correlations. Regarding focal epilepsy, whether with lesions other than hippocampal sclerosis or negative lesions, the findings revealed no significant associations with hyperthyroidism. Likewise, hereditary generalized epilepsy, juvenile myoclonic epilepsy, childhood absence epilepsy, juvenile absence epilepsy, and generalized epilepsy with tonic–clonic seizures demonstrated no significant correlations with hyperthyroidism. The robustness of these findings was established through the application of diverse analytical methods, such as the IVW method, MR-Egger method, WM method, Simple Mode method, and Weighted Mode method. Heterogeneity tests consistently indicated the absence of significant heterogeneity among instrumental variables, and sensitivity analyses, which systematically excluded SNPs, did not alter the non-significant associations. Gene pleiotropy analyses consistently revealed the absence of horizontal pleiotropy, underscoring the reliability of the results. For additional validation, an independent dataset from the FinnGen project was integrated, and the analysis confirmed the absence of significant associations between hyperthyroidism and each epilepsy subtype.

## Discussion

Jabbari and Huott reported a 9% incidence of seizures among all admissions for thyrotoxicosis. Moreover, thyrotoxicosis was identified as the primary cause of initial seizures in 1.2% of thyrotoxicosis patients admitted^27^. These findings underscore the relatively common occurrence of seizures in individuals with hyperthyroidism. Nevertheless, our understanding of the intricate mechanisms through which thyroid hormones influence brain excitability remains limited. The impact of elevated thyroid hormones on sodium–potassium adenosine triphosphatase activity, leading to significant alterations in neuronal sodium concentrations, has been acknowledged^28^. However, a comprehensive grasp of the nuanced mechanisms through which thyroid hormones modulate brain excitability remains elusive. Notably, thyrotropin-releasing hormone (TRH) has emerged as a potential therapeutic target for managing seizures in thyrotoxicosis. Experimental evidence has shown that direct hippocampal infusion of TRH produces anticonvulsant effects in amygdale-kindled rats, as evidenced by reductions in after discharge activity and seizure duration^29^. Further exploration of TRH’s role in seizure management within the context of thyrotoxicosis merits ongoing investigation.

The comprehensive etiology of epilepsy remains enigmatic; however, the roles of mitochondrial dysfunction, oxidative stress, and GABAergic system dysregulation in its progression are evident. These factors are pivotal determinants contributing to this condition. Despite the blood–brain barrier curtailing the ingress of thyroid hormones into the central nervous system, with their concentrations therein maintaining a lower equilibrium compared to serum levels, recent research has unveiled the indispensable roles of thyroid hormones in various physiological realms of the central nervous system. These functions encompass the development of the central nervous system, the sustenance of normal cerebral function, and the intricacies of reparative mechanisms. Molecular evidence substantiates the active involvement of thyroid hormones in the orchestration of normative mitochondrial biogenesis. Their deficiency is intrinsically linked to mitochondrial dysfunction and oxidative stress, both of which are held in high regard as contributory factors in the pathogenesis of epilepsy. A critical interplay between thyroid hormones and the development and function of GABAergic neurons is noteworthy. It is imperative to underscore that the unimpeded functionality of thyroid hormones is the sine qua non for the proper execution of these neurons’ functions^30^.

In contrast to prior correlation analyses based on cross-sectional studies or limited sample data, the advent of public databases has provided us with the opportunity to employ Mendelian randomization on a large scale, enabling a comprehensive examination of the causal relationship between hyperthyroidism and all forms of epilepsy. The results robustly support a causal connection between hyperthyroidism and an increased risk of various epilepsy types. This study represents the first comprehensive investigation into the association between these two conditions, shedding light on the nature and extent of their respective relationships with distinct epilepsy categories. It serves as the foundation for a more profound and precise comprehension of the interplay between these two conditions.

Mendelian randomization, a method grounded in genetic variations as instrumental variables, effectively mitigates potential confounding factors and the impacts of reverse causality. It relies on three fundamental assumptions: a robust correlation between instrumental variables and exposure, the independence of instrumental variables from the outcome, and the unrelatedness of instrumental variables to confounding factors. Consequently, this approach finds widespread application in diverse studies of disease exposure and outcomes, facilitating a more precise analysis and enhanced understanding of disease relationships^31^.

Nevertheless, this study has certain limitations: (1) Mendelian randomization assumes a linear relationship between hyperthyroidism and epilepsy. If such a linear relationship does not exist, this method may not be applicable. (2) Mendelian randomization does not delve into the biological mechanisms underlying the association between hyperthyroidism and epilepsy. (3) Insufficient data, including age and gender, are available, which hinders in-depth analysis.

The present study is not without limitations. Firstly, in the Mendelian randomization (MR) analysis of the relationship between hyperthyroidism and epilepsy, the sample size is insufficient, resulting in lower statistical power. Although no anomalies were detected in other sensitivity analyses, this limitation compromises the robustness of the results, necessitating future investigations in larger databases and clinical studies to elucidate their relationship. Secondly, all participants in the Genome-Wide Association Studies (GWAS) datasets are of European descent, raising questions about the generalizability of the results to other populations, warranting further research. Thirdly, thyroid dysfunction is significantly influenced by gender, with the incidence in females being 8 to 9 times that in males^32^. However, the twin-sample design employed in this study did not stratify by gender and age, precluding an analysis of the relationship between hyperthyroidism and epilepsy across different genders and age groups. Lastly, although the GWAS data used in this study pertain to hyperthyroidism (congenital or acquired), a detailed stratification analysis for congenital and acquired hyperthyroidism was not conducted, demanding further comprehensive research to validate our findings.

## Conclusions

This study found evidence for a possible link between hyperthyroidism and an increased risk of epilepsy.

### Supplementary Information

Supplementary Information.

## Data Availability

The data originates from the IEU OpenGWAS Project (specific URL detailed in Supplementary Table S1) and the Finn Gen database (specific URL detailed in Supplementary Table S13); for further inquiries, please contact the corresponding author.
