# Supplementary material for: Associations of hyperthyroidism with epilepsy: a Mendelian randomization study
Source: Sci Rep. 2024 Feb 27;14:4733. doi: 10.1038/s41598-024-54933-w (PMC10899576; doi:10.1038/s41598-024-54933-w)
Supplement: Supplementary file 1 — Supplementary Information. [file 41598_2024_54933_MOESM1_ESM.zip › Supplementary.docx]

**
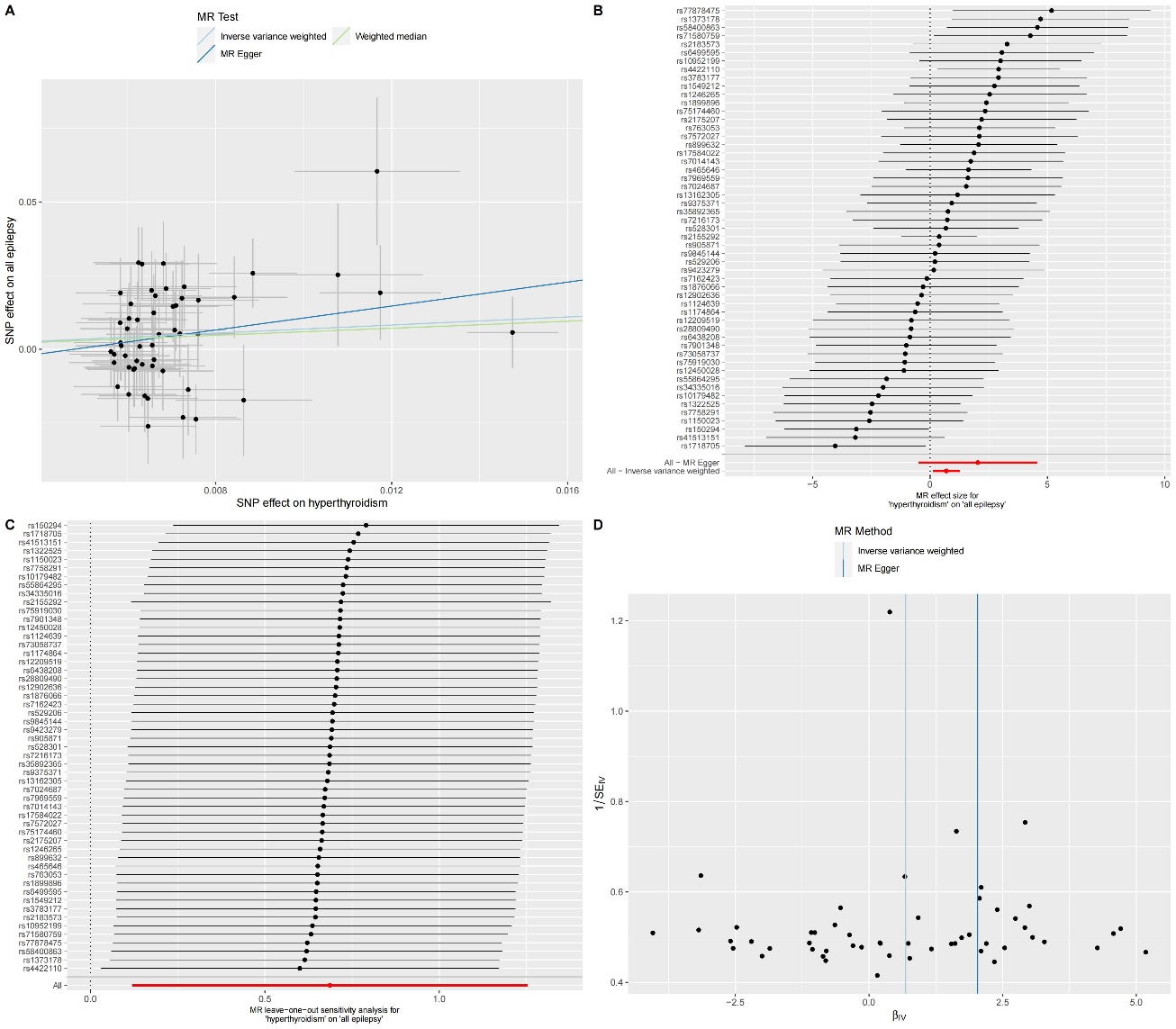
Supplementary Figure 1.** Scatter plot (A), forest plot (B), and “leave-one-out” analysis (C) for MR analysis of hyperthyroidism(IEU) and all epilepsy, funnel plot (D).


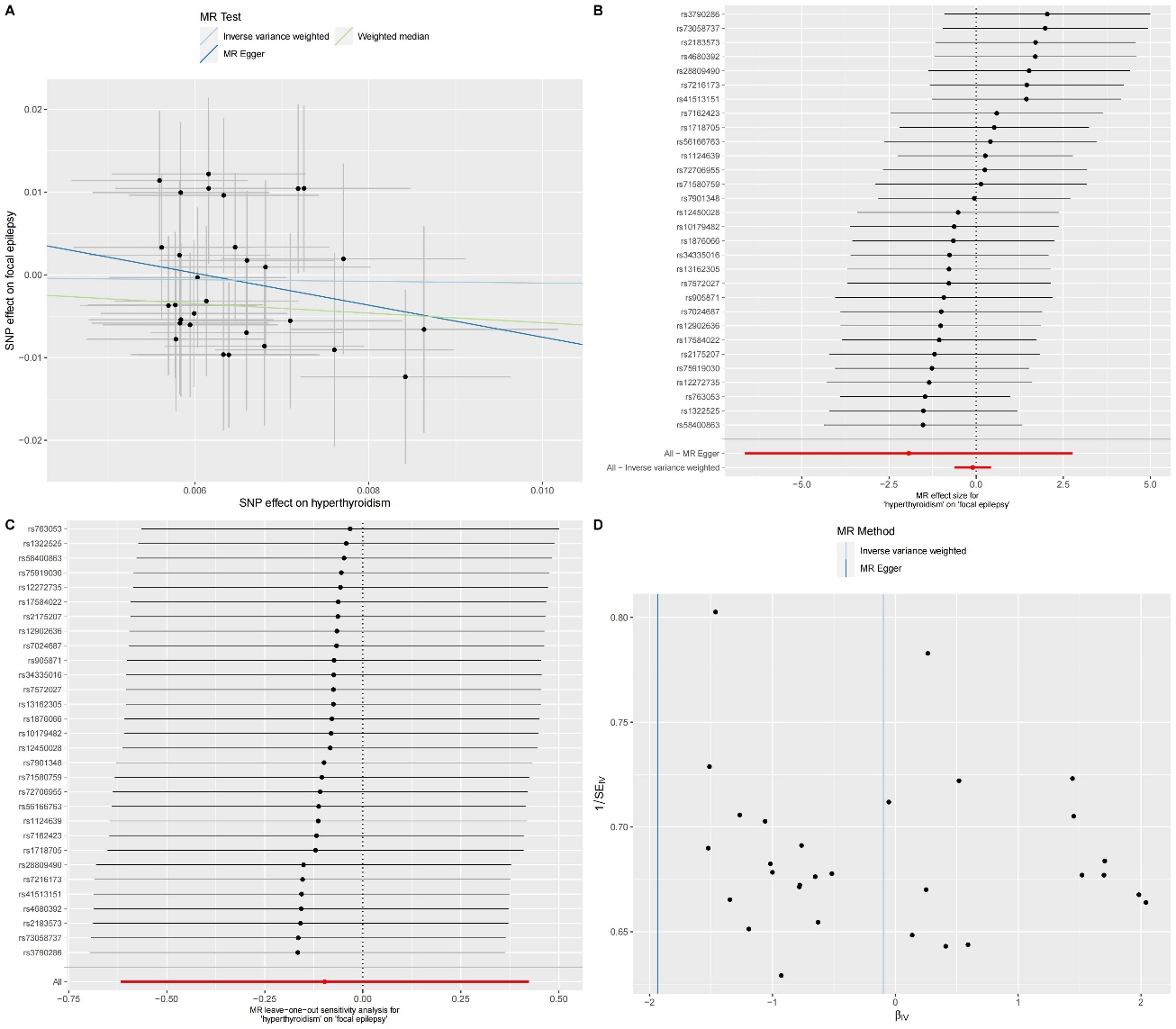


**Supplementary Figure 2.** Scatter plot (A), forest plot (B), and “leave-one-out” analysis (C) for MR analysis of hyperthyroidism(IEU) and focal epilepsy, funnel plot (D).


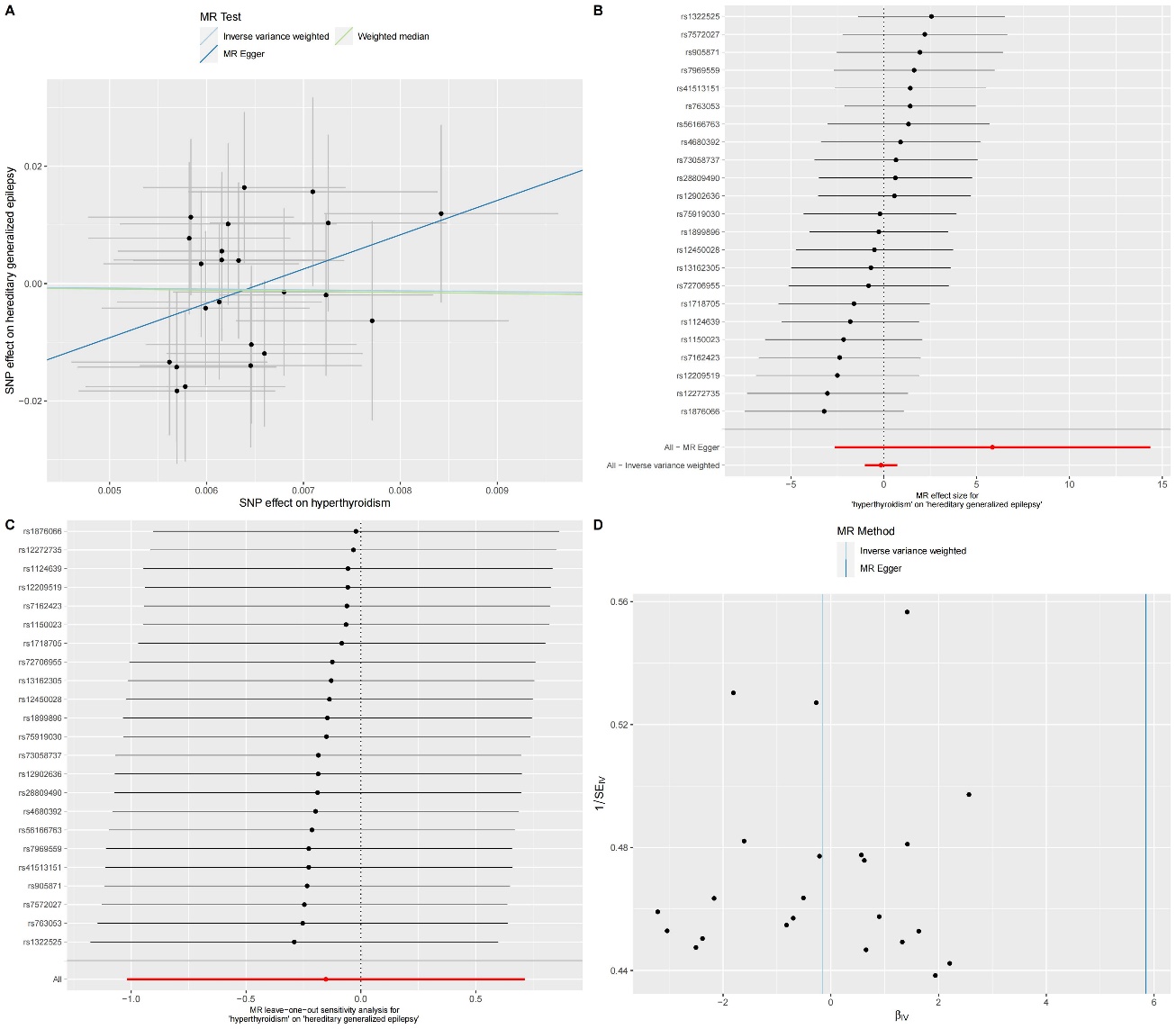


**Supplementary Figure 3.** Scatter plot (A), forest plot (B), and “leave-one-out” analysis (C) for MR analysis of hyperthyroidism(IEU) and hereditary generalized epilepsy, funnel plot (D).


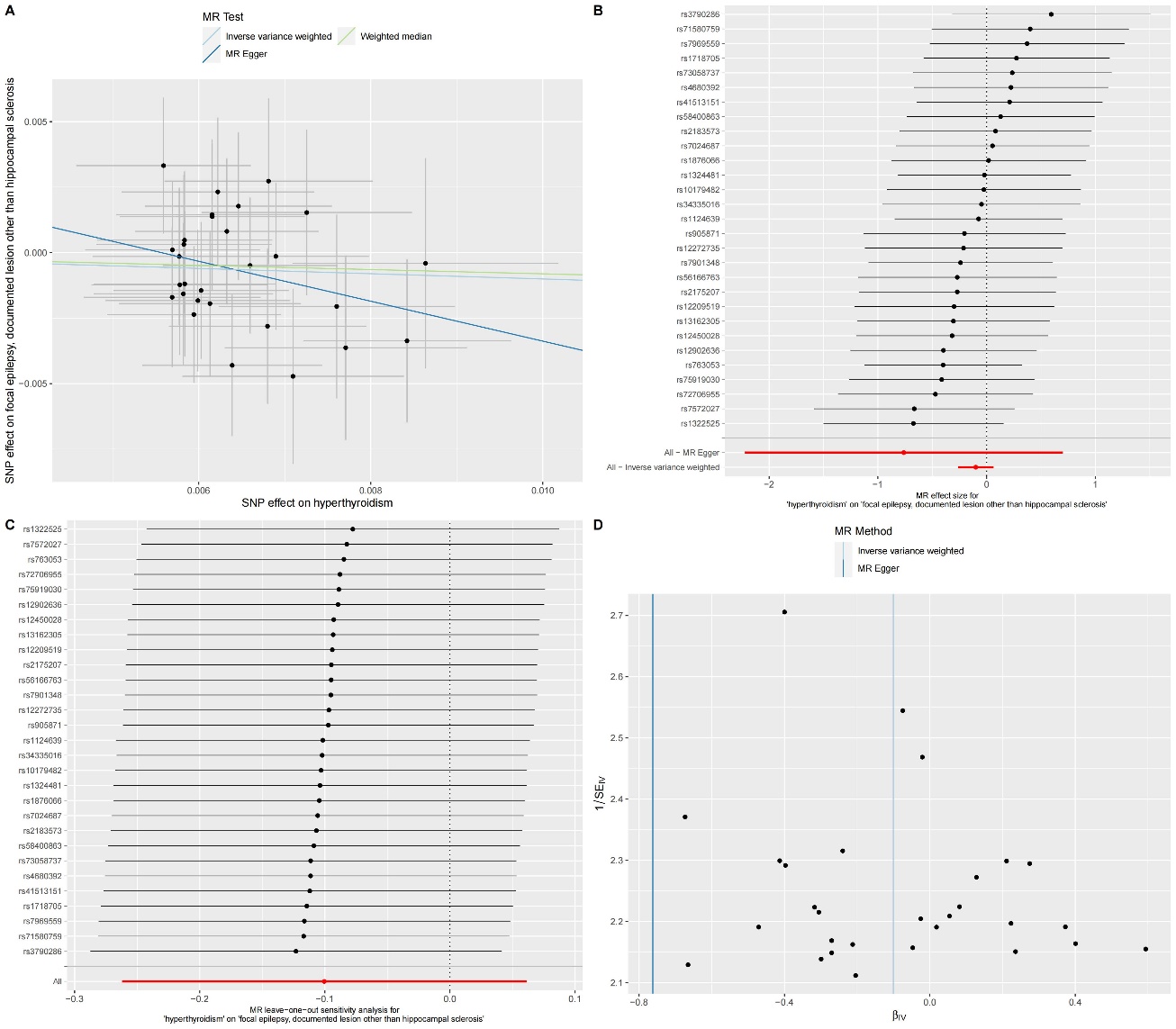


**Supplementary Figure 4.** Scatter plot (A), forest plot (B), and “leave-one-out” analysis (C) for MR analysis of hyperthyroidism(IEU) and focal epilepsy (documented lesion other than hippocampal sclerosis), funnel plot (D).

**
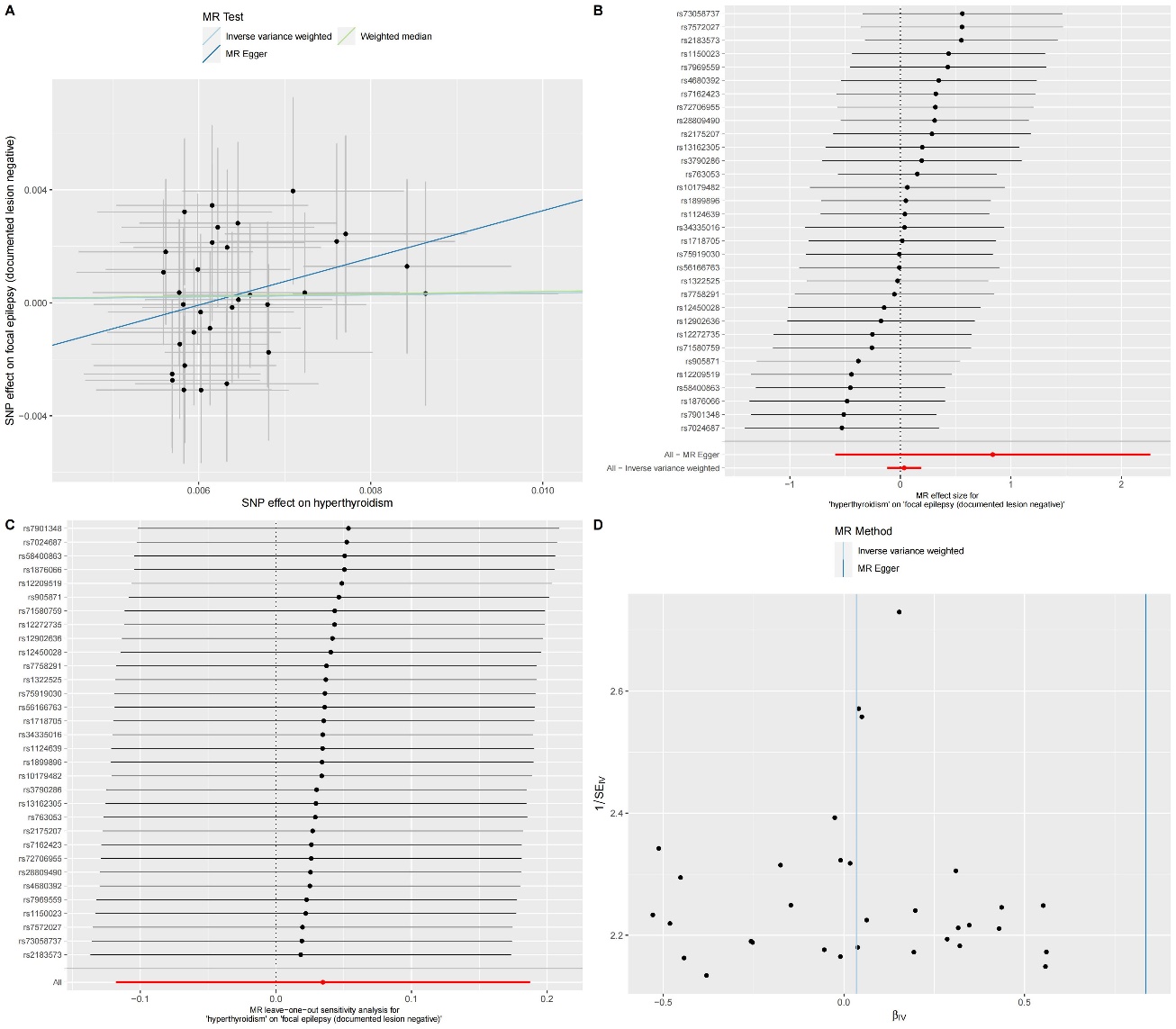
Supplementary Figure 5.** Scatter plot (A), forest plot (B), and “leave-one-out” analysis (C) for MR analysis of hyperthyroidism(IEU) and focal epilepsy (documented lesion negative), funnel plot (D).

**Supplementary Figure 6.** Scatter plot (A), forest plot (B), and “leave-one-out” analysis (C) for MR analysis of hyperthyroidism(IEU) and juvenile myoclonic epilepsy, funnel plot (D).
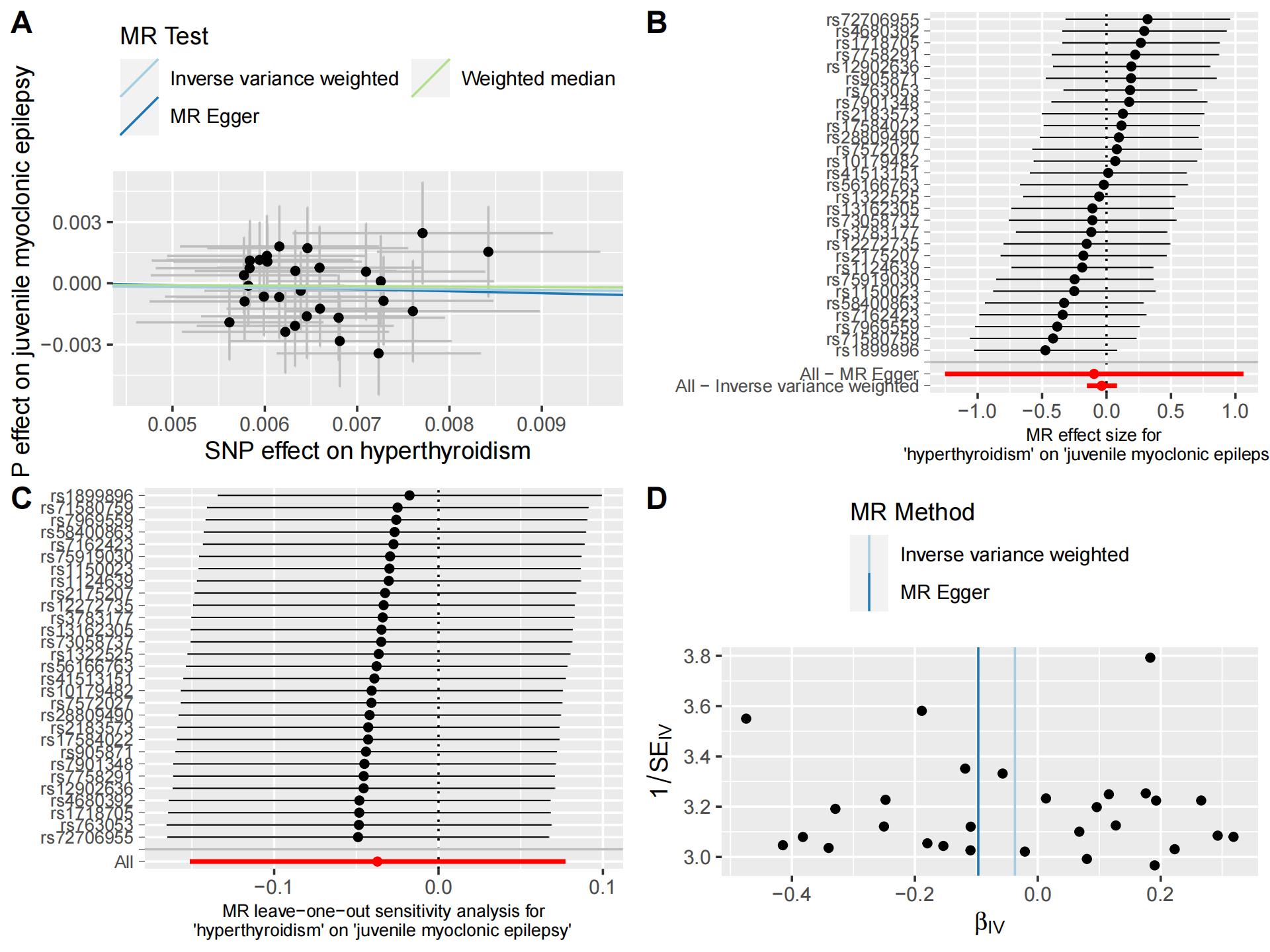


**
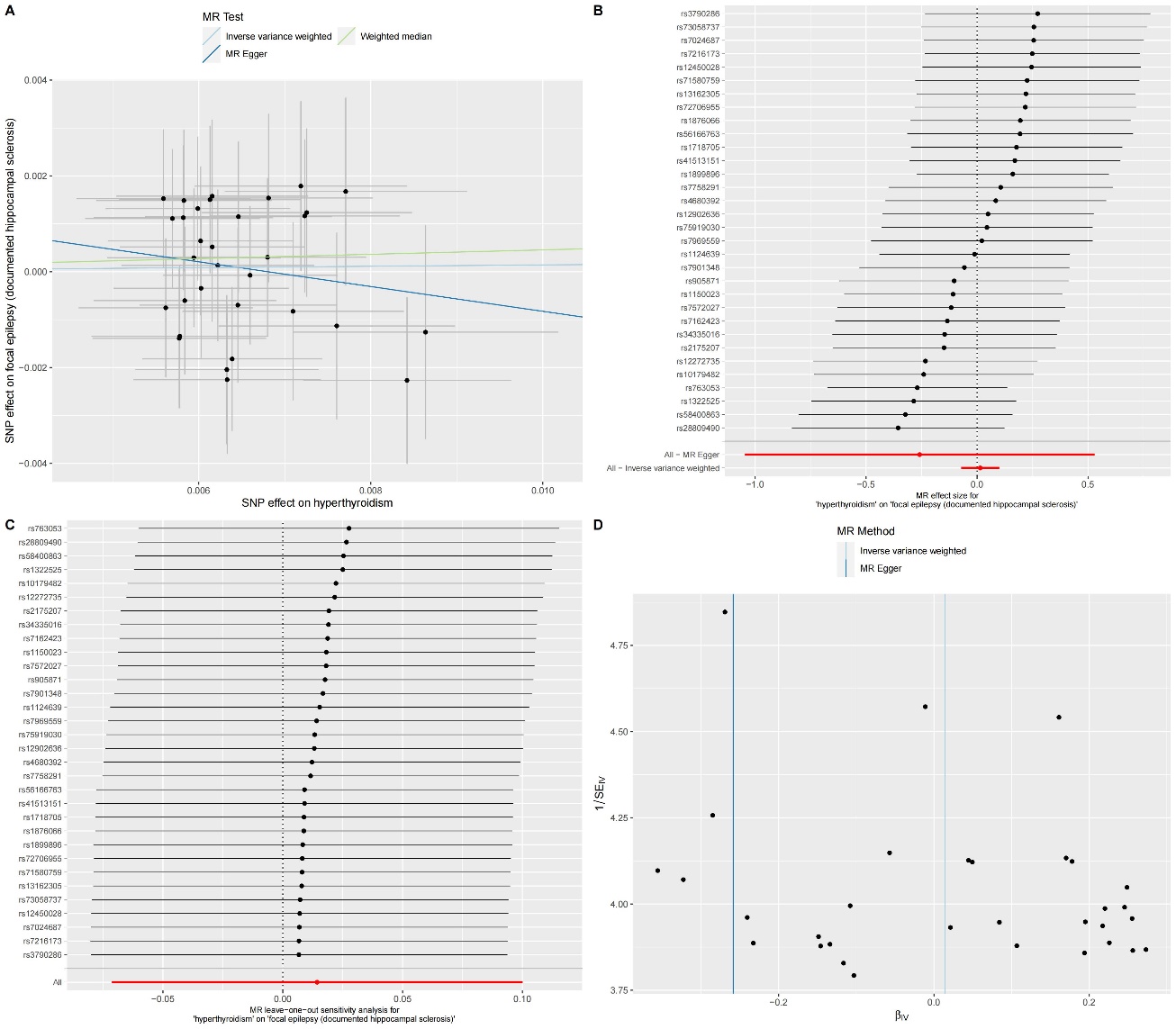
Supplementary Figure 7.** Scatter plot (A), forest plot (B), and “leave-one-out” analysis (C) for MR analysis of hyperthyroidism(IEU) and focal epilepsy (documented hippocampal sclerosis), funnel plot (D).

**Supplementary Figure 8.** Scatter plot (A), forest plot (B), and “leave-one-out” analysis (C) for MR analysis of hyperthyroidism(IEU) and childhood absence epilepsy, funnel plot (D).
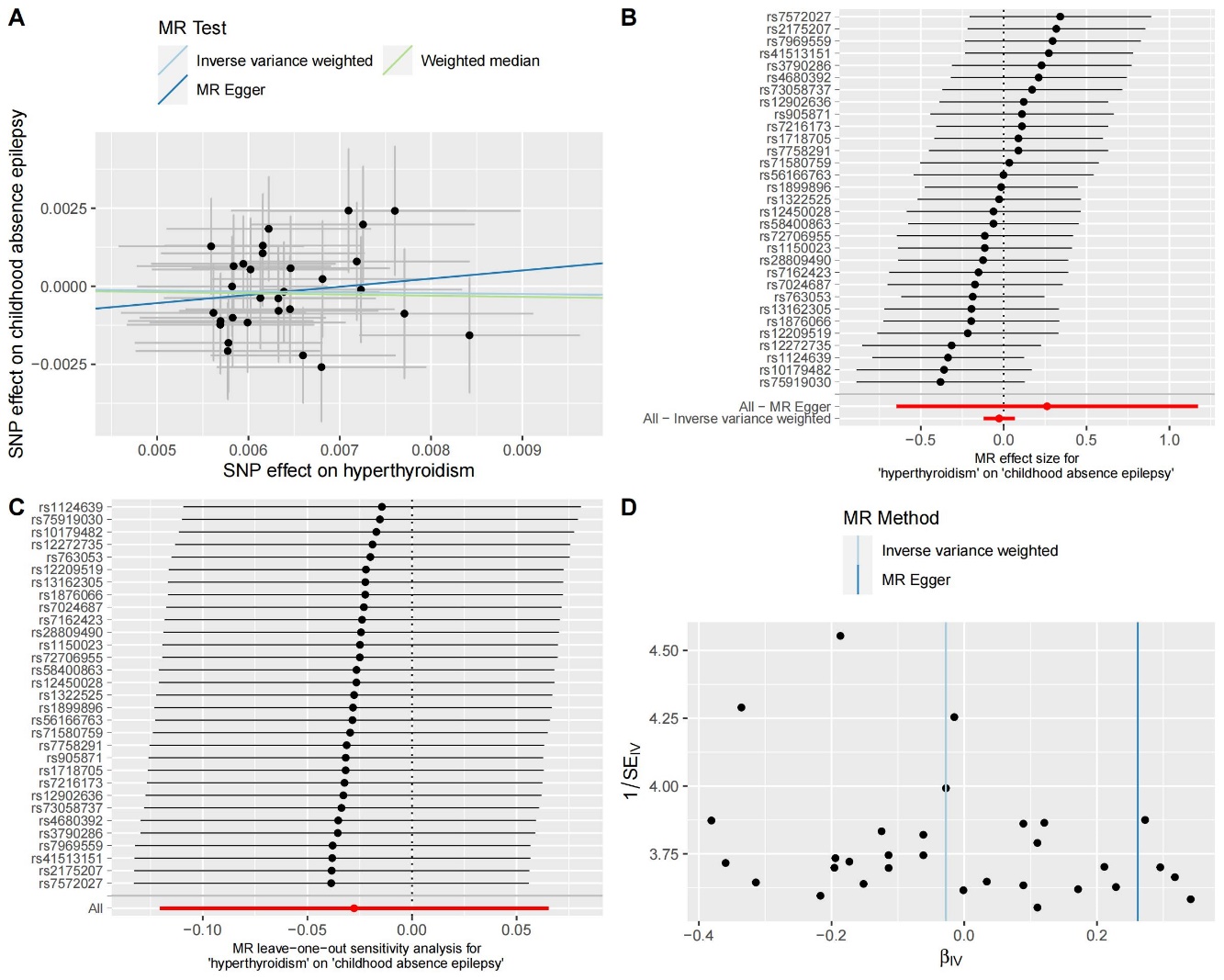


**
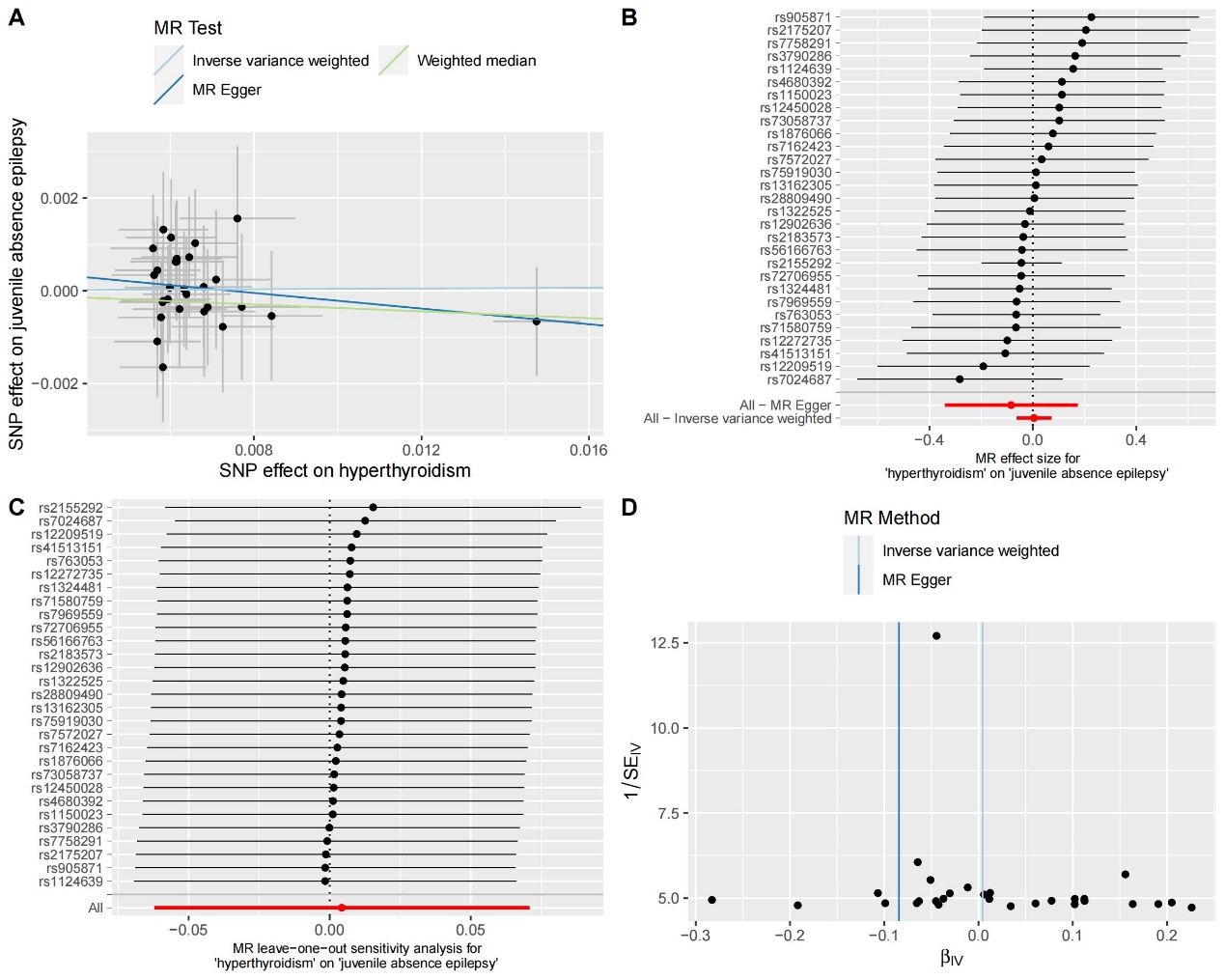
Supplementary Figure 9.** Scatter plot (A), forest plot (B), and “leave-one-out” analysis (C) for MR analysis of hyperthyroidism(IEU) and juvenile absence epilepsy, funnel plot (D).
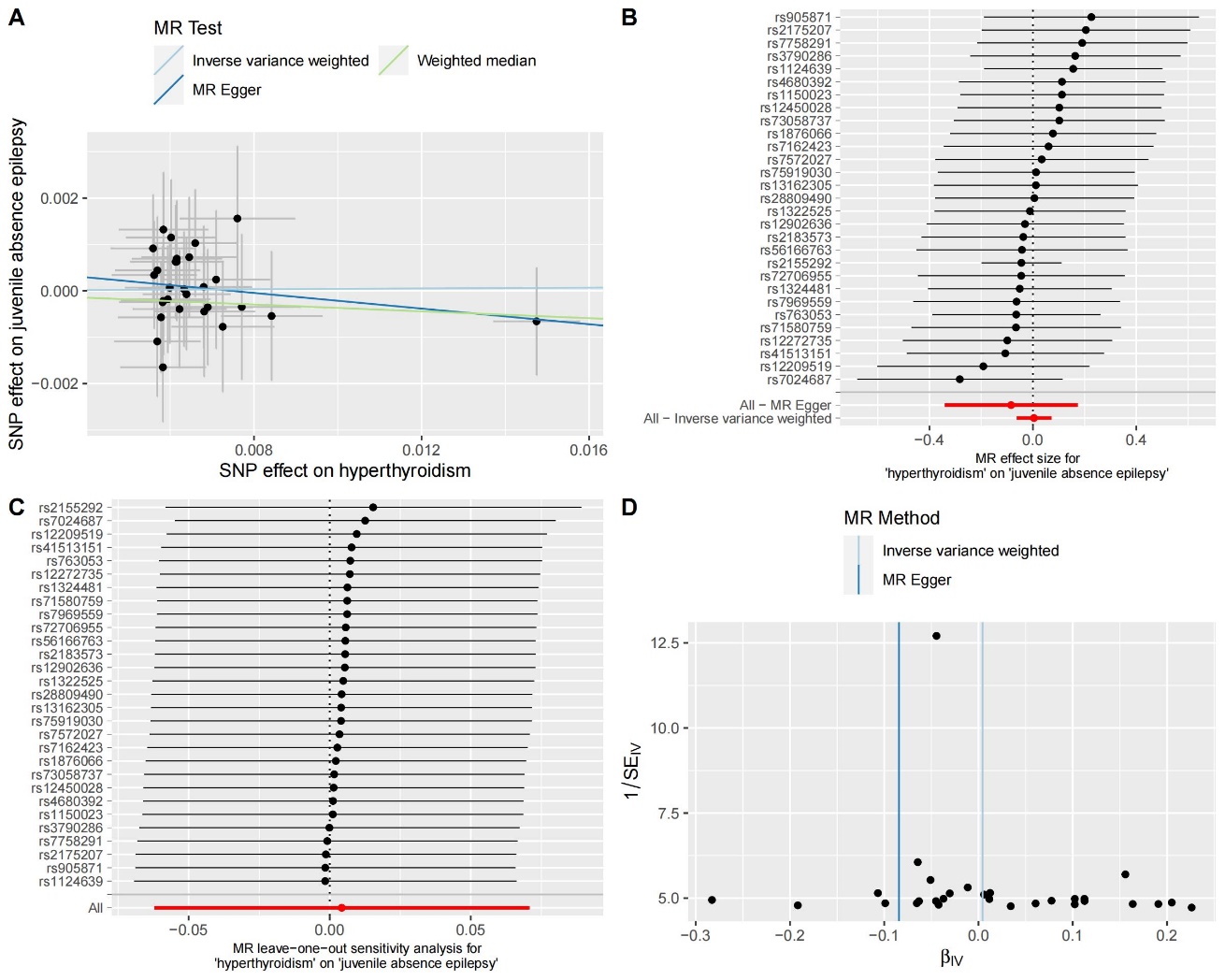
**Supplementary Figure 10.** Scatter plot (A), forest plot (B), and “leave-one-out” analysis (C) for MR analysis of hyperthyroidism(IEU) and generalized epilepsy with tonic-clonic seizures, funnel plot (D).


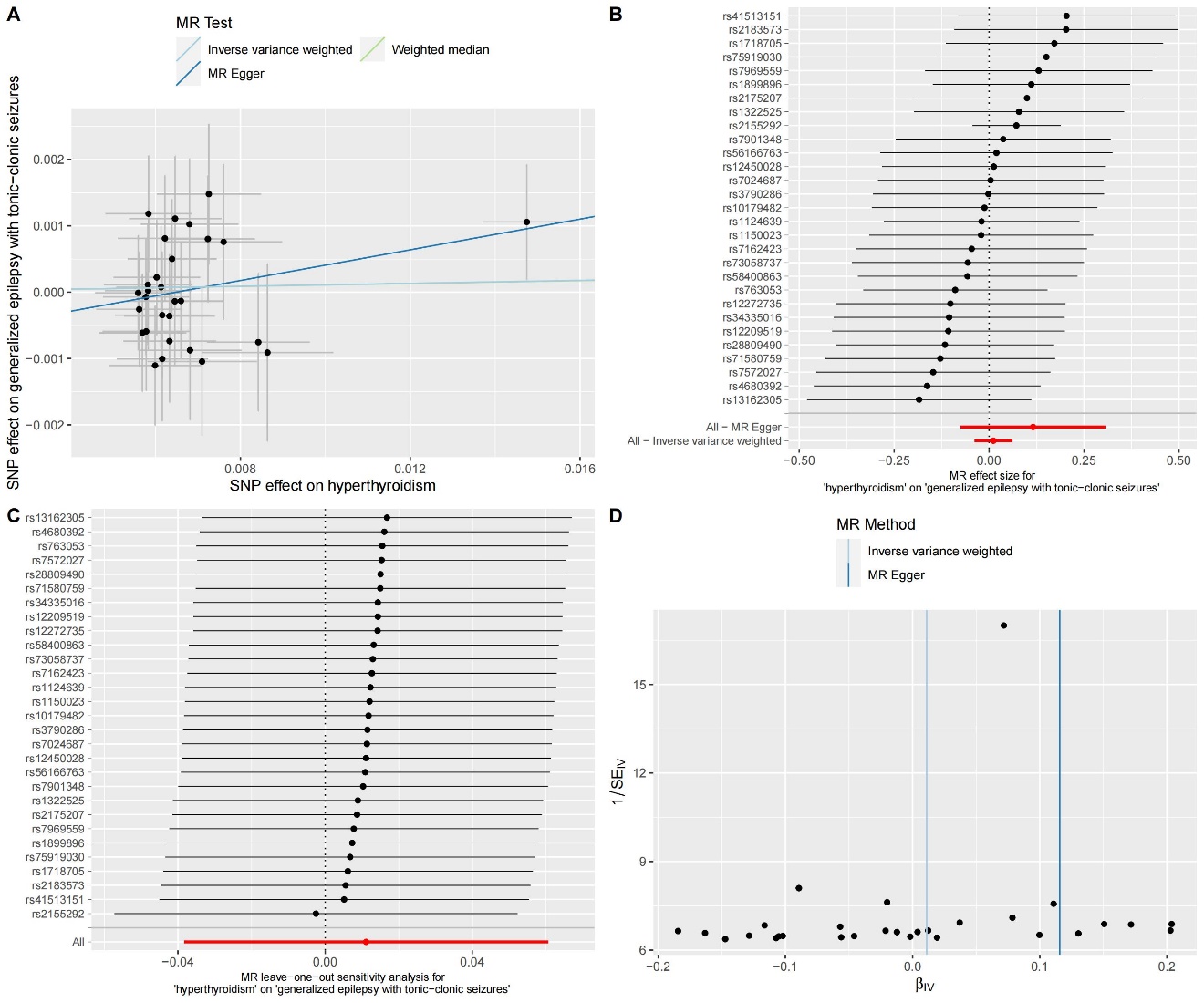


**Supplementary Figure 11.** Scatter plot (A), forest plot (B), and “leave-one-out” analysis (C) for MR analysis of hyperthyroidism(Finngen) and all epilepsy, funnel plot (D).**
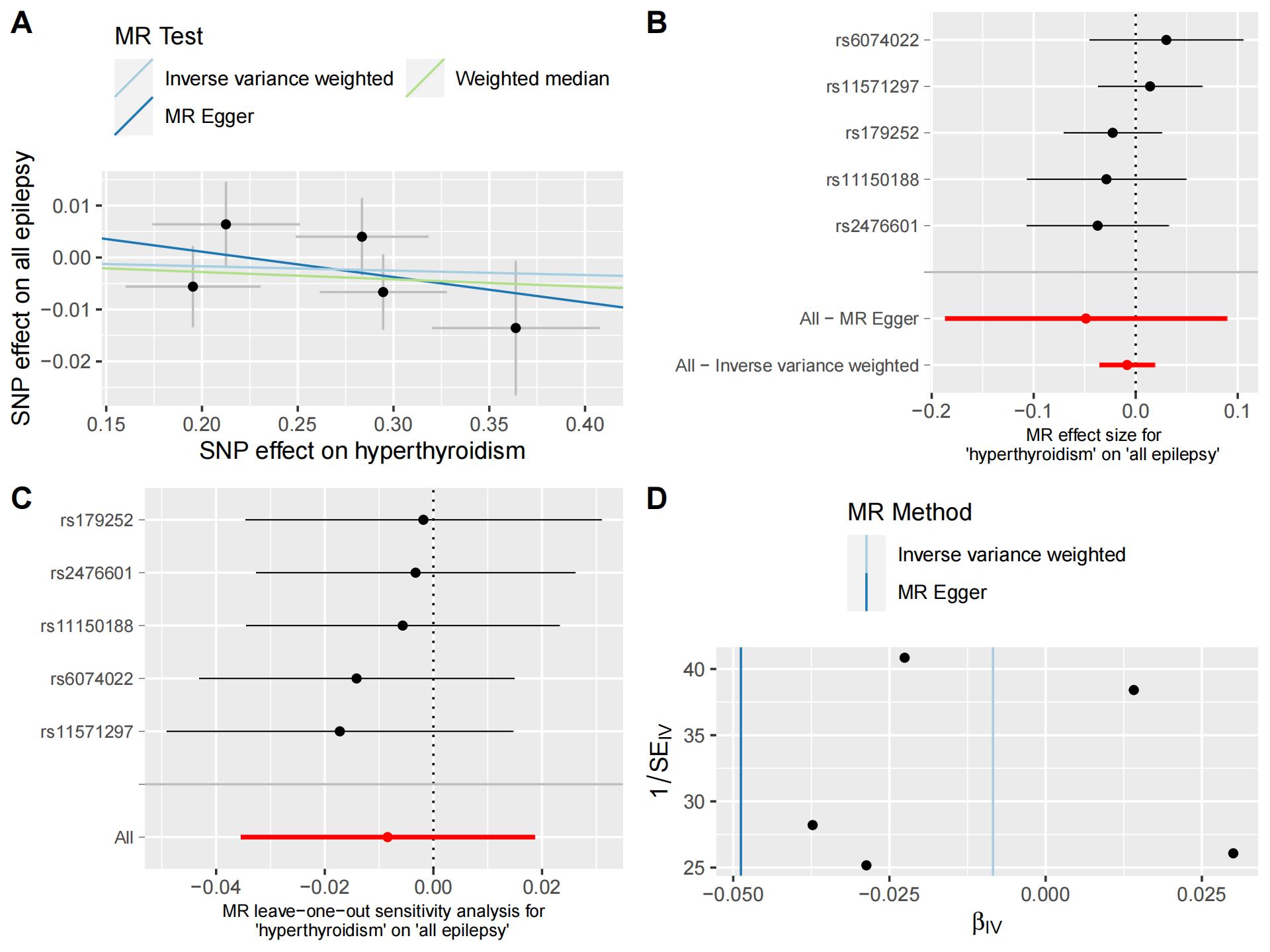
**

**Supplementary Figure 12.** Scatter plot (A), forest plot (B), and “leave-one-out” analysis (C) for MR analysis of hyperthyroidism(Finngen) and focal epilepsy, funnel plot (D).


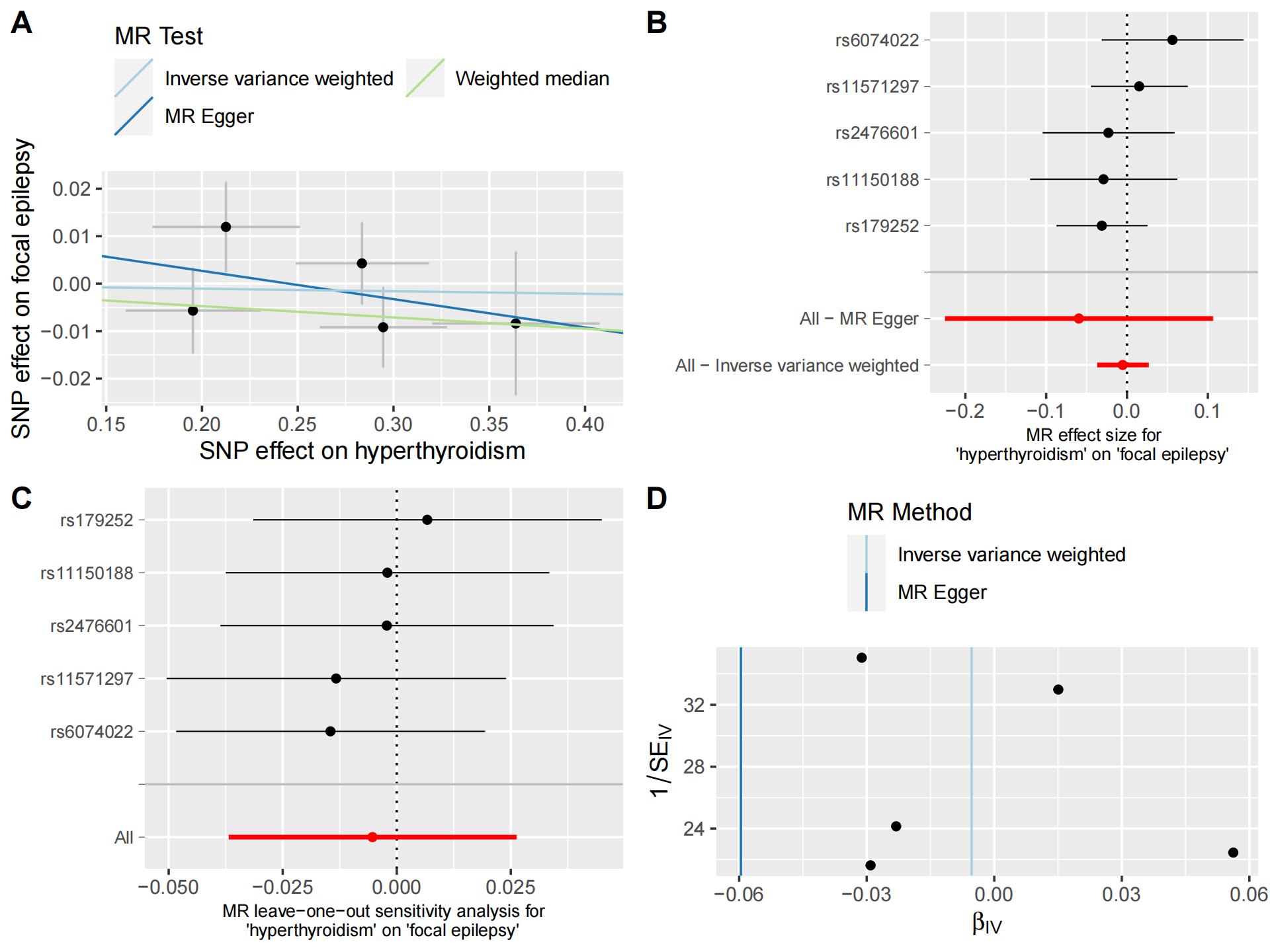


**Supplementary Figure 13.** Scatter plot (A), forest plot (B), and “leave-one-out” analysis (C) for MR analysis of hyperthyroidism(Finngen) and hereditary generalized epilepsy, funnel plot (D).
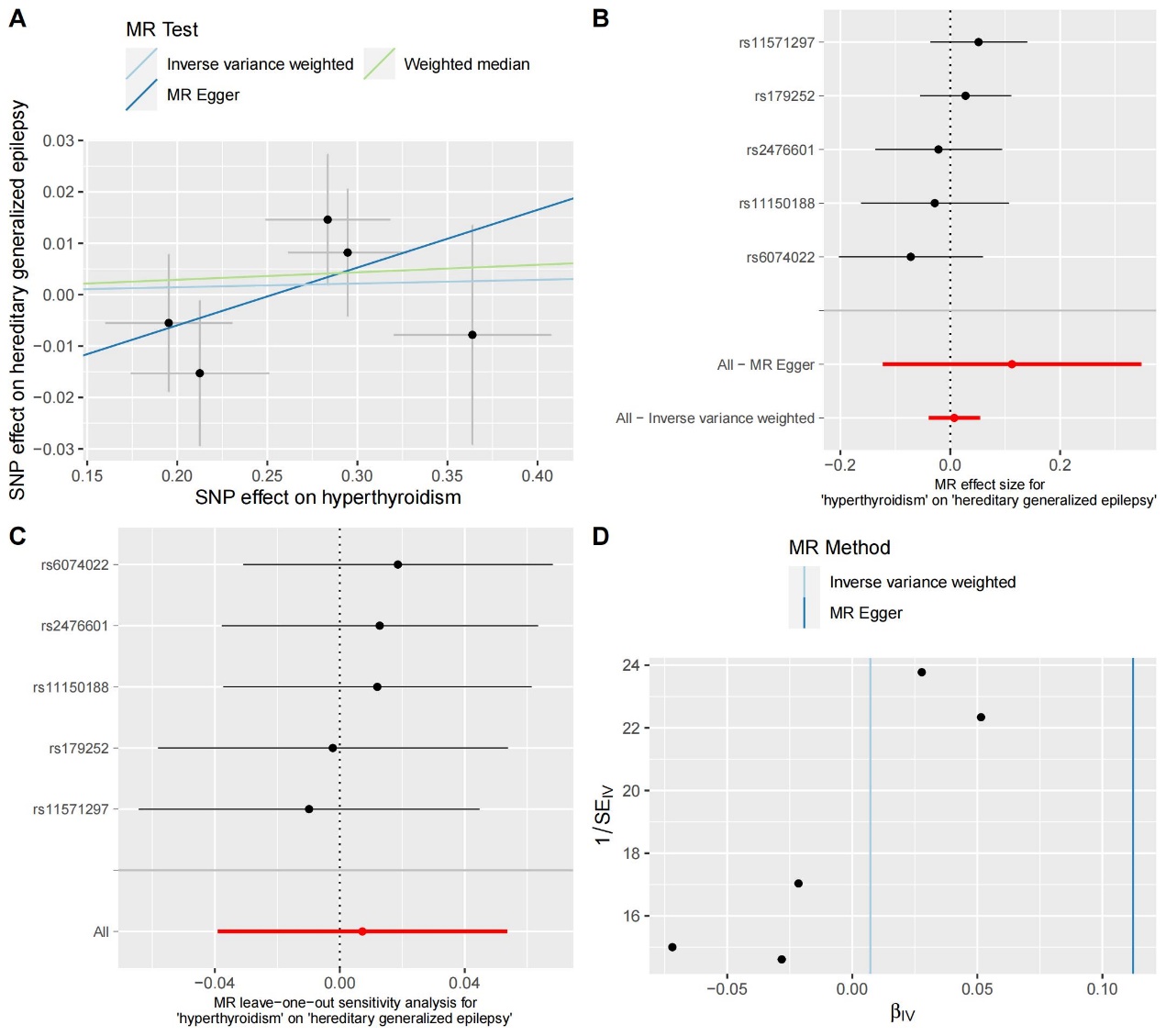


**Supplementary Figure 14.** Scatter plot (A), forest plot (B), and “leave-one-out” analysis (C) for MR analysis of hyperthyroidism(Finngen) and focal epilepsy (documented lesion other than hippocampal sclerosis), funnel plot (D).
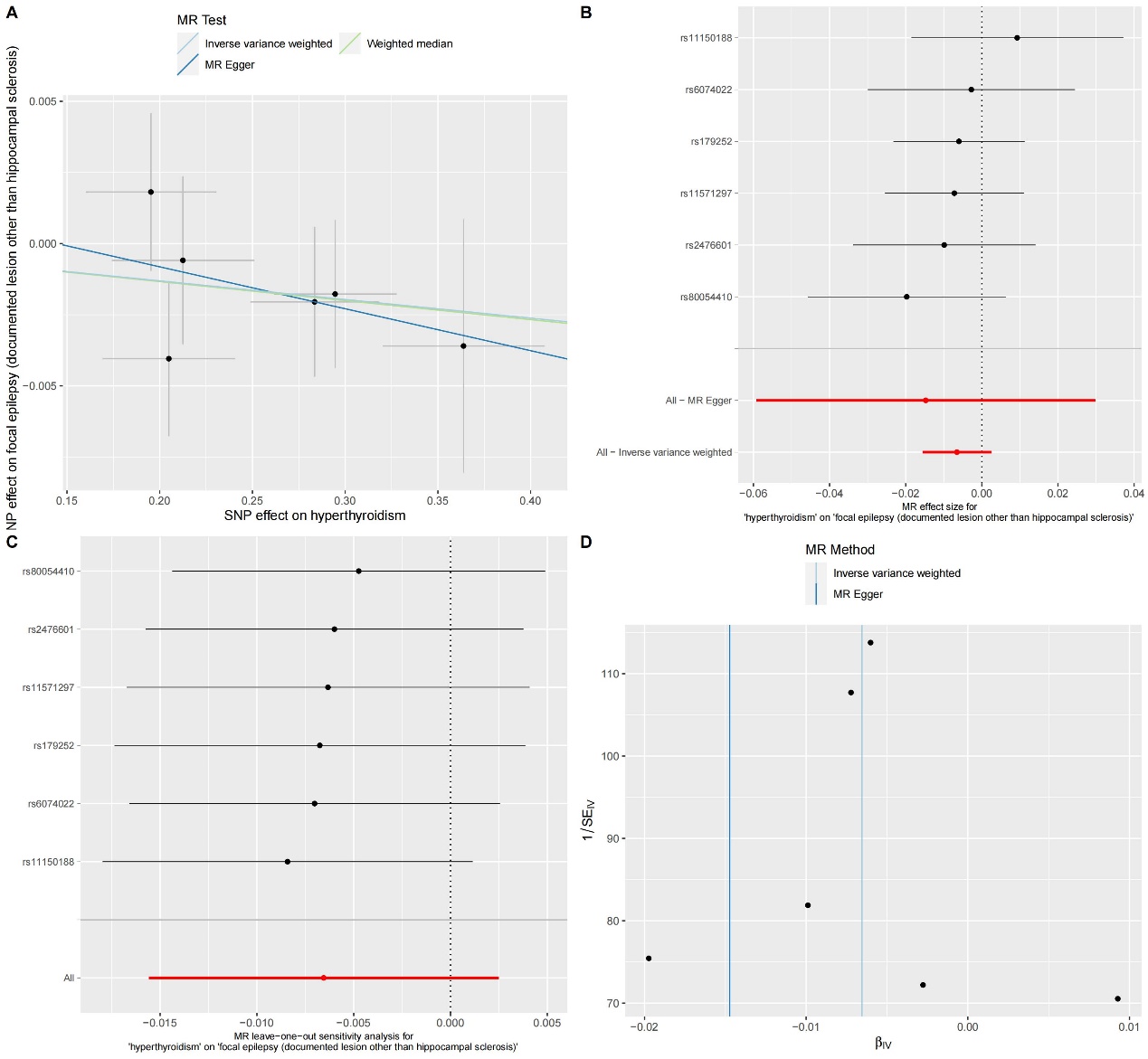


**Supplementary Figure 15.** Scatter plot (A), forest plot (B), and “leave-one-out” analysis (C) for MR analysis of hyperthyroidism(Finngen) and focal epilepsy (documented lesion negative), funnel plot (D).

**
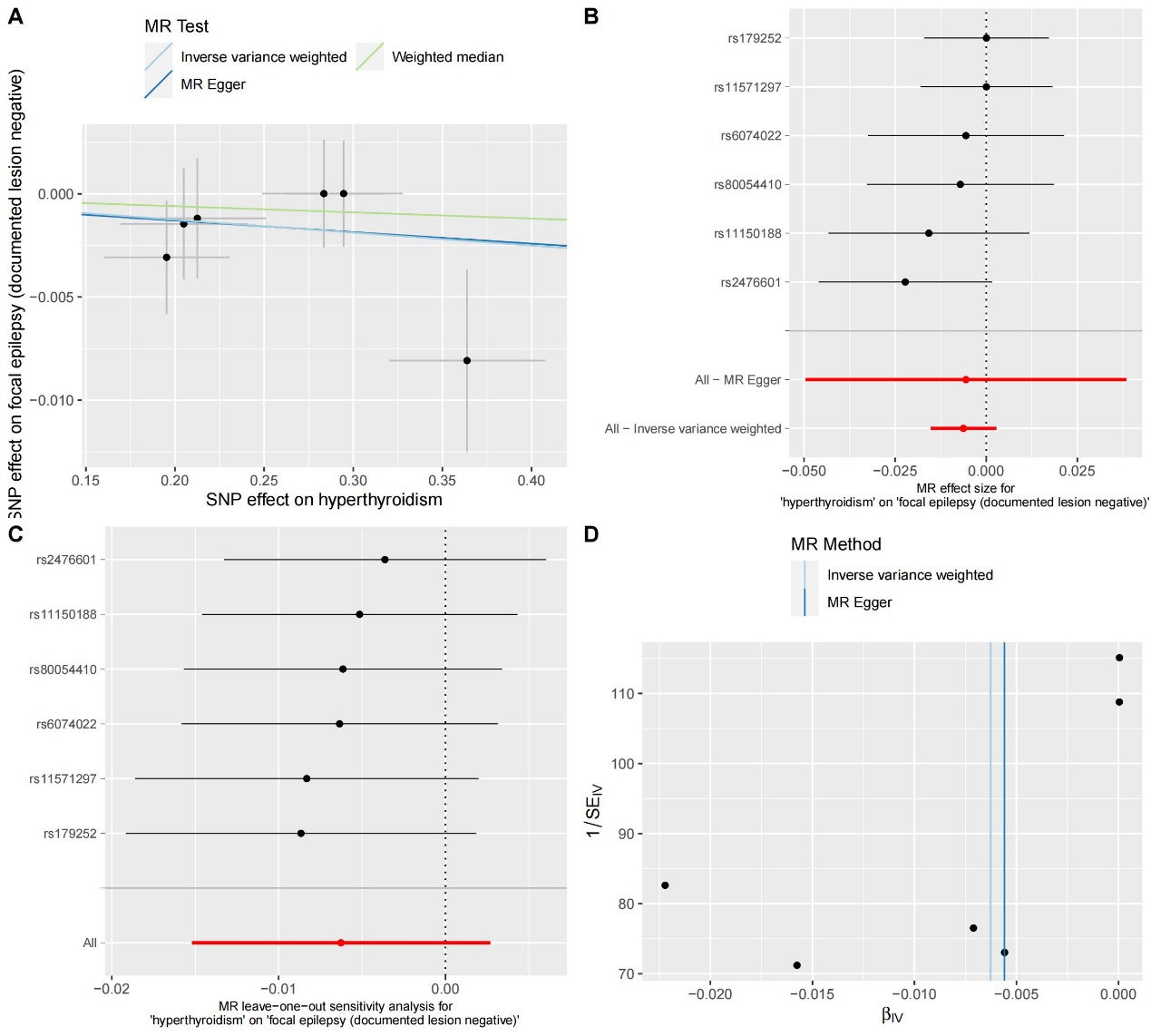
**

**Supplementary Figure 16.** Scatter plot (A), forest plot (B), and “leave-one-out” analysis (C) for MR analysis of hyperthyroidism(Finngen) and juvenile myoclonic epilepsy, funnel plot (D).**
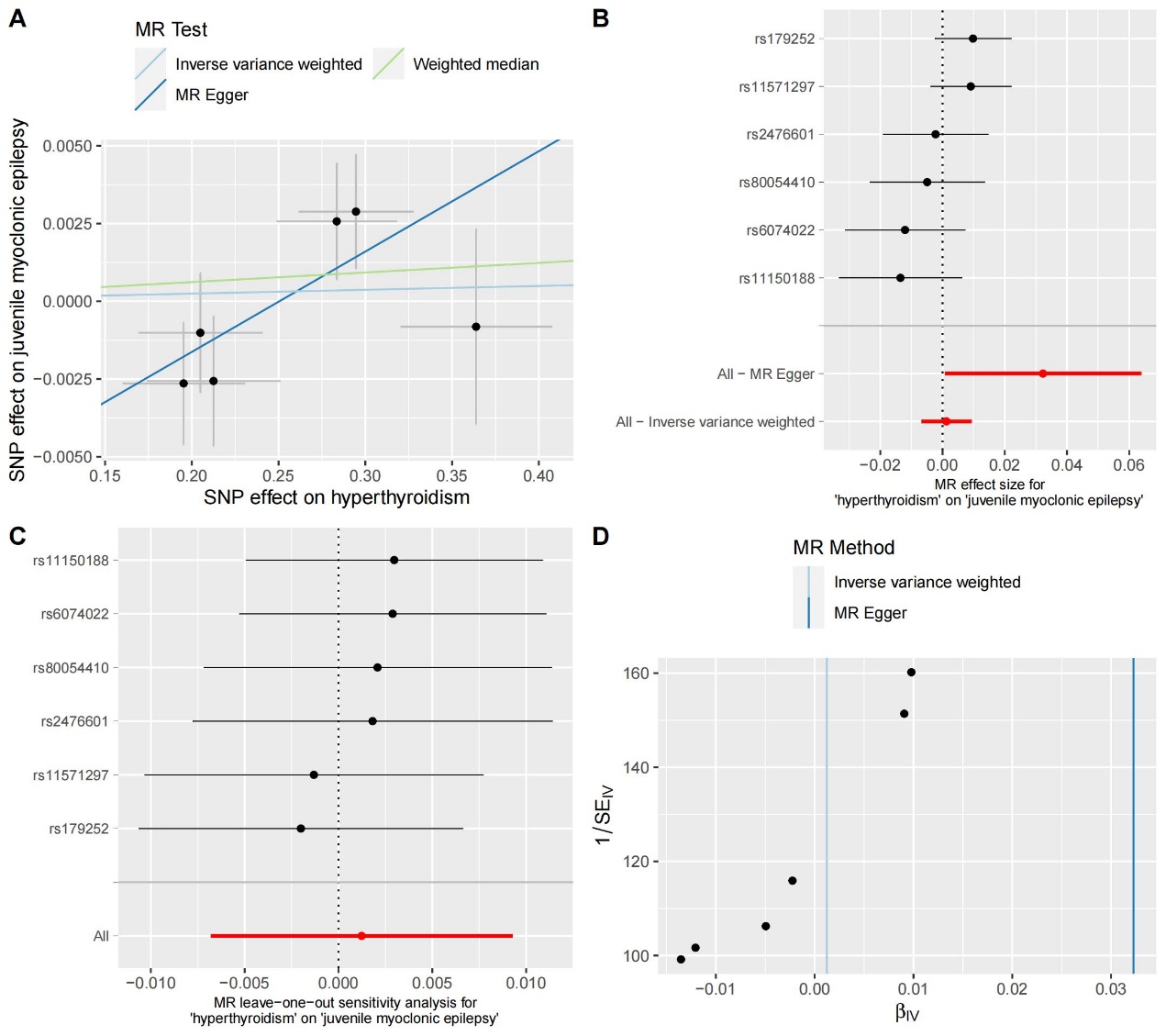
**

**Supplementary Figure 17.** Scatter plot (A), forest plot (B), and “leave-one-out” analysis (C) for MR analysis of hyperthyroidism(Finngen) and focal epilepsy (documented hippocampal sclerosis), funnel plot (D).**
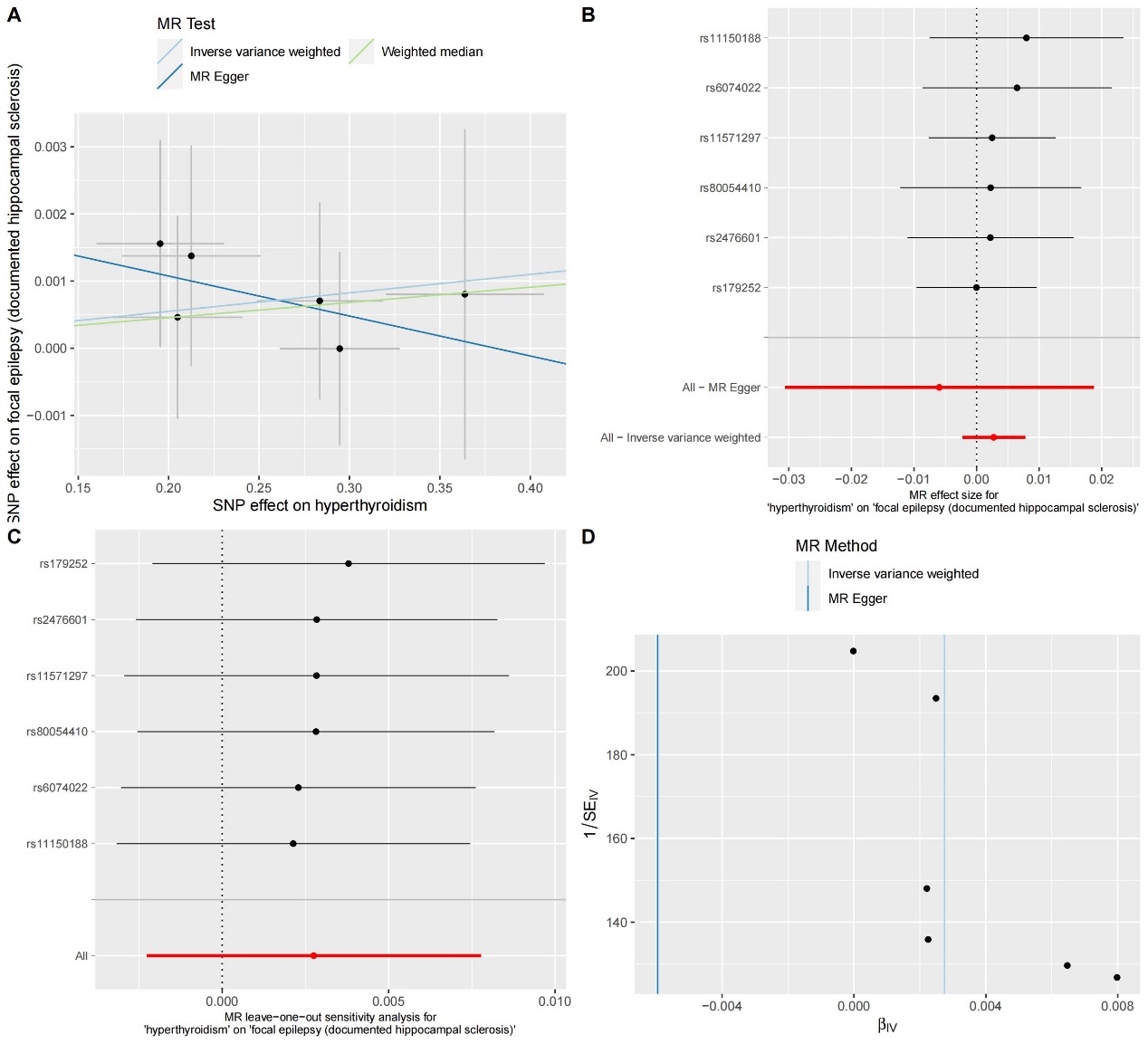
**

**Supplementary Figure 18.** Scatter plot (A), forest plot (B), and “leave-one-out” analysis (C) for MR analysis of hyperthyroidism(Finngen) and childhood absence epilepsy, funnel plot (D).**
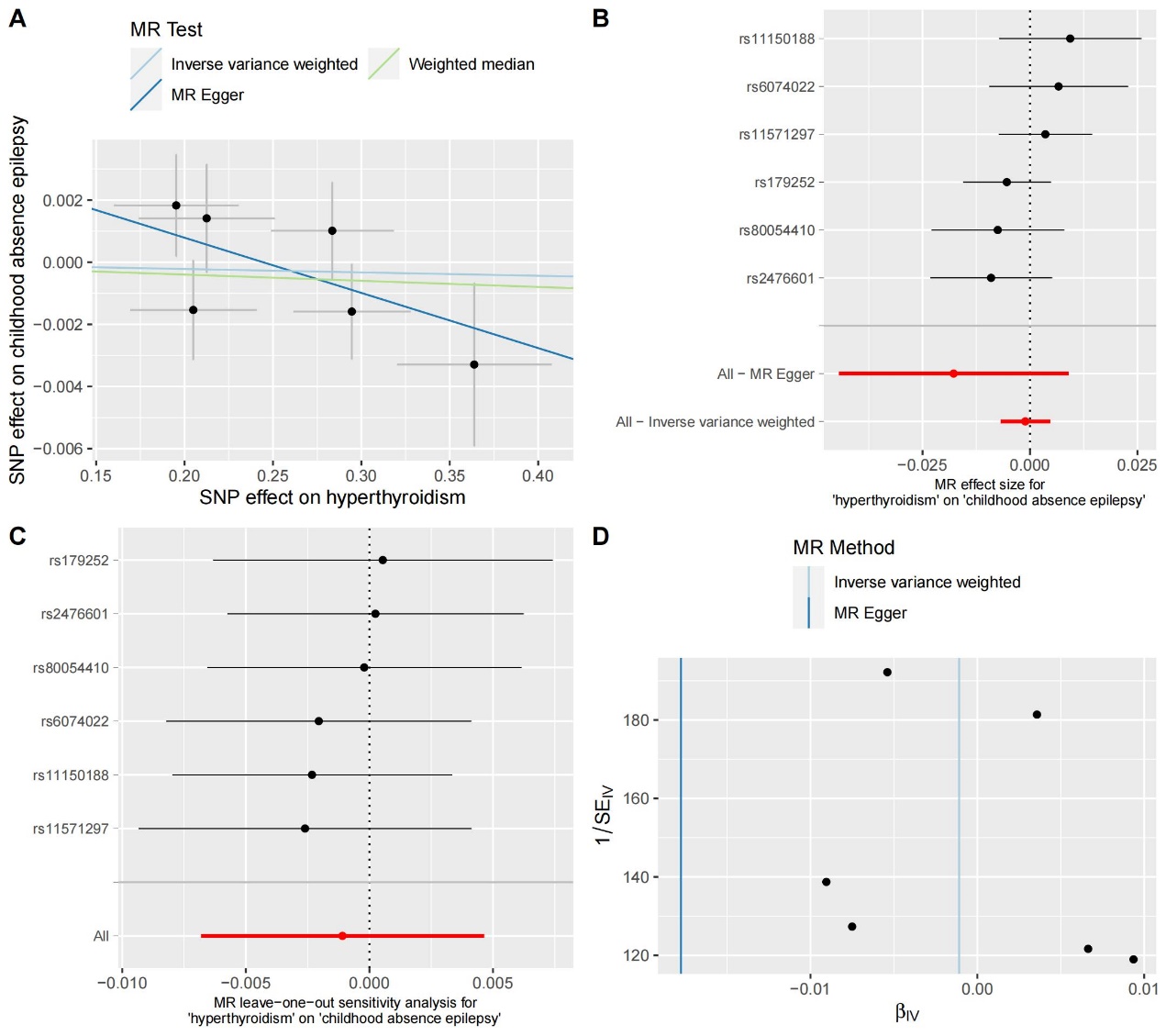
**

**Supplementary Figure 19.** Scatter plot (A), forest plot (B), and “leave-one-out” analysis (C) for MR analysis of hyperthyroidism(Finngen) and juvenile absence epilepsy, funnel plot (D).**
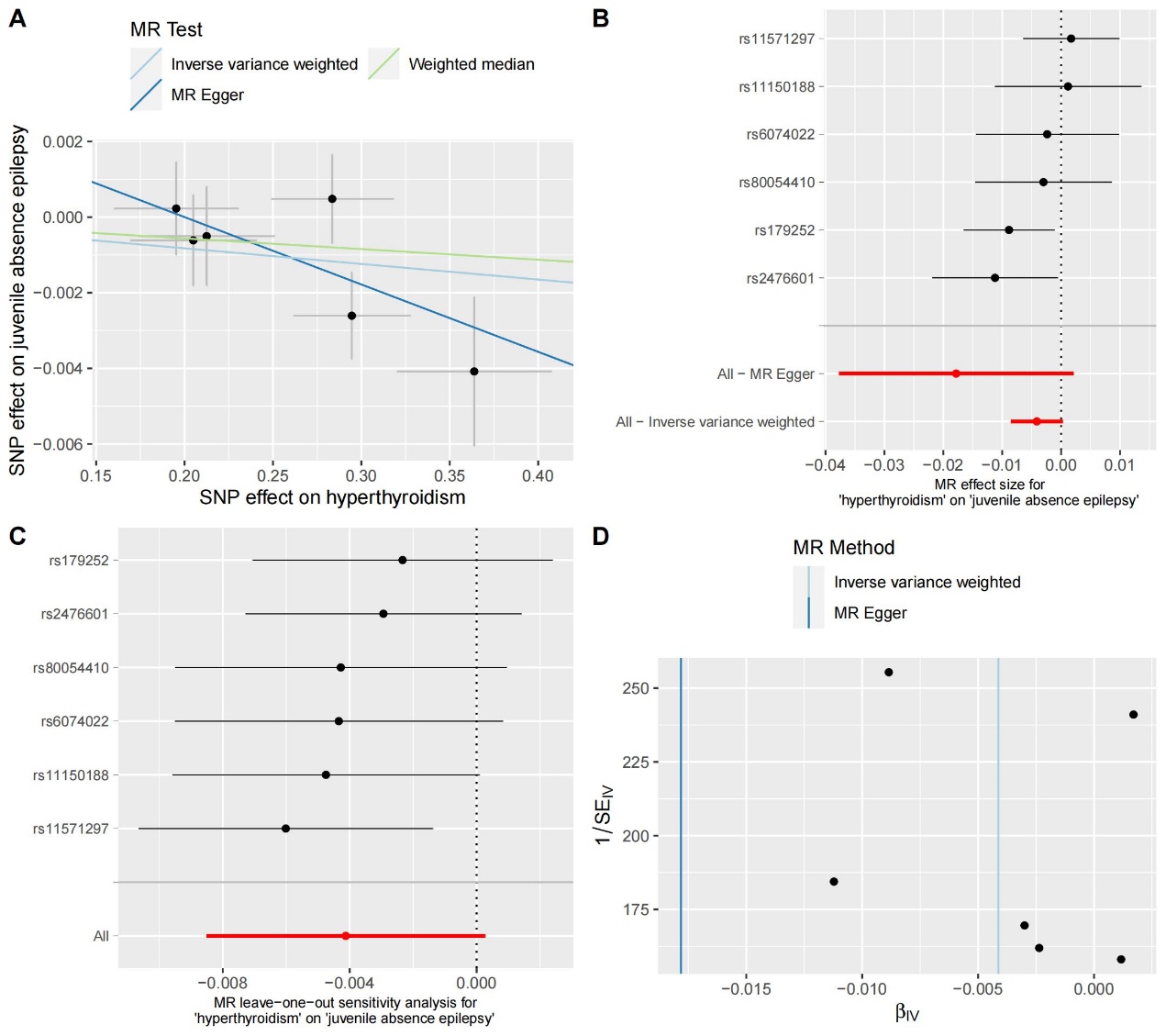
Supplementary Figure 20.** Scatter plot (A), forest plot (B), and “leave-one-out” analysis (C) for MR analysis of hyperthyroidism(Finngen) and generalized epilepsy with tonic-clonic seizures, funnel plot (D).

**
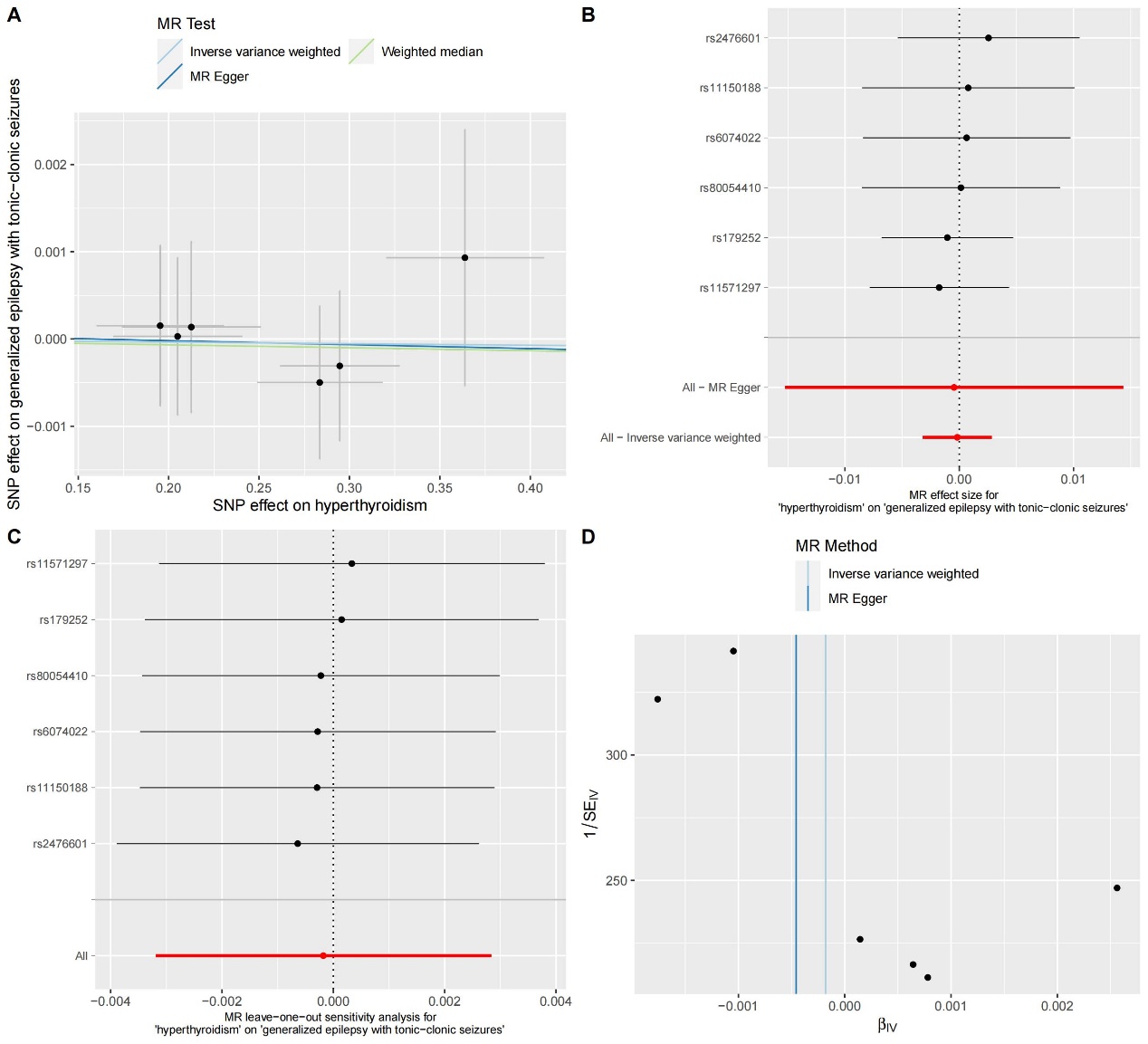
**
